# High impact health service interventions for attainment of UHC in Africa: A systematic review

**DOI:** 10.1371/journal.pgph.0000945

**Published:** 2022-09-23

**Authors:** Humphrey Cyprian Karamagi, Araia Berhane, Solyana Ngusbrhan Kidane, Lizah Nyawira, Mary Ani-Amponsah, Loise Nyanjau, Koulthoume Maoulana, Aminata Binetou Wahebine Seydi, Jacinta Nzinga, Jean-marie Dangou, Triphonie Nkurunziza, Geoffrey K. Bisoborwa, Jackson Sophianu Sillah, Assumpta W. Muriithi, Harilala Nirina Razakasoa, Francoise Bigirimana

**Affiliations:** 1 Data Analytics and Knowledge Management, World Health Organization (WHO) Regional Office for Africa, Brazzaville, Republic of Congo; 2 Conmmunicable Diseases Control Division, Ministry of Health, Asmara, Eritrea; 3 Health Economics Research Unit, KEMRI-Wellcome Trust Research Programme, Nairobi, Kenya; 4 University of Ghana, Accra, Ghana; 5 National Cancer Institute of Kenya, Nairobi, Kenya; 6 Ministry of Health, Solidarity, Social Protection and Gender Promotion, Moroni, Comoros; 7 Health Services Unit, KEMRI-Wellcome Trust Research Programme, Nairobi, Kenya; 8 Non-communicable Disease, WHO AFRO, Brazzaville, Congo; 9 Reproductive and Maternal Health, WHO AFRO, Brazzaville, Congo; 10 Child and Adolescent Health, WHO AFRO, Brazzaville, Congo; 11 Tropical and Vector Born Disease, WHO AFRO, Brazzaville, Congo; 12 HIV, TB and Hepatitis, WHO AFRO, Brazzaville, Congo; University of the Witwatersrand, SOUTH AFRICA

## Abstract

African countries have prioritized the attainment of targets relating to Universal Health Coverage (UHC), Health Security (HSE) and Coverage of Health Determinants (CHD)to attain their health goals. Given resource constraints, it is important to prioritize implementation of health service interventions with the highest impact. This is important to be identified across age cohorts and public health functions of health promotion, disease prevention, diagnostics, curative, rehabilitative and palliative interventions. We therefore explored the published evidence on the effectiveness of existing health service interventions addressing the diseases and conditions of concern in the Africa Region, for each age cohort and the public health functions. Six public health and economic evaluation databases, reports and grey literature were searched. A total of 151 studies and 357 interventions were identified across different health program areas, public health functions and age cohorts. Of the studies, most were carried out in the African region (43.5%), on communicable diseases (50.6%), and non-communicable diseases (36.4%). Majority of interventions are domiciled in the health promotion, disease prevention and curative functions, covering all age cohorts though the elderly cohort was least represented. Neonatal and communicable conditions dominated disease burden in the early years of life and non-communicable conditions in the later years. A menu of health interventions that are most effective at averting disease and conditions of concern across life course in the African region is therefore consolidated. These represent a comprehensive evidence-based set of interventions for prioritization by decision makers to attain desired health goals. At a country level, we also identify principles for identifying priority interventions, being the targeting of higher implementation coverage of existing interventions, combining interventions across all the public health functions–not focusing on a few functions, provision of subsidies or free interventions and prioritizing early identification of high-risk populations and communities represent these principles.

## Introduction

As countries in Africa pursue the way for attainment of the sustainable development goals and universal health coverage, there is a need to re-pivot health systems, identify the diseases and conditions that cause the primary country-specific Disability-Adjusted Life Years (DALYs) and divert their limited resources to interventions that result in most lives saved and disabilities averted. These high impact interventions categorised as activities or actions undertaken at an individual, community or programmatic levels are expected to improve the health condition of individuals or communities by preventing infections or diseases, cure diseases or suppressing disease-causing germs/viruses or reducing the severity or duration of an existing disease, or by restoring function lost due to diseases or injuries [1]. Effective or high impact interventions, implemented strategically at the right time for the right population with high coverage, can eliminate conditions that cause the majority of morbidities, mortalities and disabilities. Cost-effectiveness has been one of the criteria used by WHO CHOICE [2] and Disease Control Priorities 3^rd^ edition (DCP-3) [3] in prioritization of interventions for the recommended essential health care packages. The proof of effectiveness in averting DALYs for major conditions in the region could be an additional impetus for countries in prioritizing interventions when compiling their packages.

With the global advancement of science, technology and human innovations, several high-impact interventions of low cost but proven effectiveness have been implemented across regions. These interventions have reduced the burden of diseases and conditions globally and in the African Region. Of particular interest are interventions that contributed to the reduction in communicable diseases. A good example is malaria interventions that resulted in marked reduction in communicable diseases within the region. As a result of the global concerted effort and implementation of high impact interventions of insecticide treated bed nets, indoor residual spraying and artesunate based case management, an estimated 1.7 billion malaria cases and 10.6 million malaria deaths with 85% and 95%, respectively in the WHO Africa region, were averted in the last two decades (2000–2020) [4]. Equally, low-cost and effective interventions also exist for reducing the risks and diseases related to non-communicable diseases (NCDs) [5] and Reproductive Maternal Neonatal, Child and Adolescent Health (RMNCAH) conditions [6]. If implemented across all public health functions and age cohorts, these interventions could help countries achieve a significant reduction in morbidities and mortalities across populations. Even more critical is how the evidence on the effectiveness of these interventions can provide countries with guidance on how they can re-pivot their health system development efforts towards attaining the results expected of them. The question then is centred on which diseases constitute the current burden of disease and what interventions are most effective in addressing them? Among the myriad of diseases that afflict the population in the region, some are more common in some specific age groups than others and contribute to DALYs more than others [7]; similarly, some interventions are also more effective than others.

Therefore, it is essential to have a common platform against which the effectiveness of different interventions can be compared to ensure countries can make the best decisions about what to focus on to reduce cost and maximize health outcomes. DALY estimates allow comparability between the impact of diseases and potential benefit of proposed measures set against similar and comparable data of other health problems as it aggregates the total health loss at population level into a single index by summarising years of life lost due to premature death (YLLs) and years lived with disability (YLDs) [7, 8]. Therefore, this comprehensive review is aimed at consolidating the effectiveness of various interventions across public health functions in averting the DALYs contributed by the leading communicable & NCDs, maternal and neonatal conditions and injuries in the different age cohorts in the African Region. This review is intended to influence and shape policies, practices and service delivery, while advancing the road to UHC in the continent.

## Methodology

### Measures of disease burden

Before compiling the interventions, the leading causes of DALYs in the African region were identified and ranked for each age cohort (new born/neonate, under five, 5–9, 10–24, 25–59, 60+. The primary source for this task was the data base of the Institute for Health Metrics and Evaluation (IHME) 2019 [9], which provided information on incidence, DALYs, and deaths for different age groups over extended period of time. Based on this analysis five leading causes of DALYs for each age cohort were prioritized for identifying appropriate interventions. All data on disease burden extracted from the IHME database and are attached in the supplementary appendix **S1 Data**.

### Measures of effectiveness

This review employed the DALY as the measure of effectiveness for all identified interventions for the preventive, promotive, curative and rehabilitative/palliative public health functions. In the rehabilitation and palliative care setting, Quality Adjusted Life Years (QALYs) gained was used as additional measure of effectiveness.

### Inclusion and exclusion criteria

Articles that were published i) in English or French ii) between 2000–2022 iii) included all types of interventions with health effects expressed in DALY or QALY (specifically for rehabilitative and palliative care interventions) iv) limited to the diseases and conditions identified as major contributors of DALY in the Africa region v) stratified across age cohorts and public health functions vi) all countries were included.

Articles i) languages was not English or French ii) publication date was before the year 2000 iii) different outcome measures from those in our inclusion criteria iv) interventions targeted conditions beyond the determined high burden contributors vi) study protocols, opinions and comments were excluded.

### Search strategy

A comprehensive search strategy adopting key search terms on the subject area based on the PICO framework was conducted. Search terms included “specific age cohorts”, “specific interventions”, “outcome measures of interest” and relevant synonyms. Medical subject headings (MeSH) and key text words were developed and combined with Boolean operators “AND” and/ “OR” across and within categories as necessary. A filter was applied to restrict the type of studies to systematic reviews, meta-analysis, randomized controlled trials, case-control studies or cohort studies. The entire search strategy for the PubMed database was tested and adapted to other databases.

**Fig 1:**
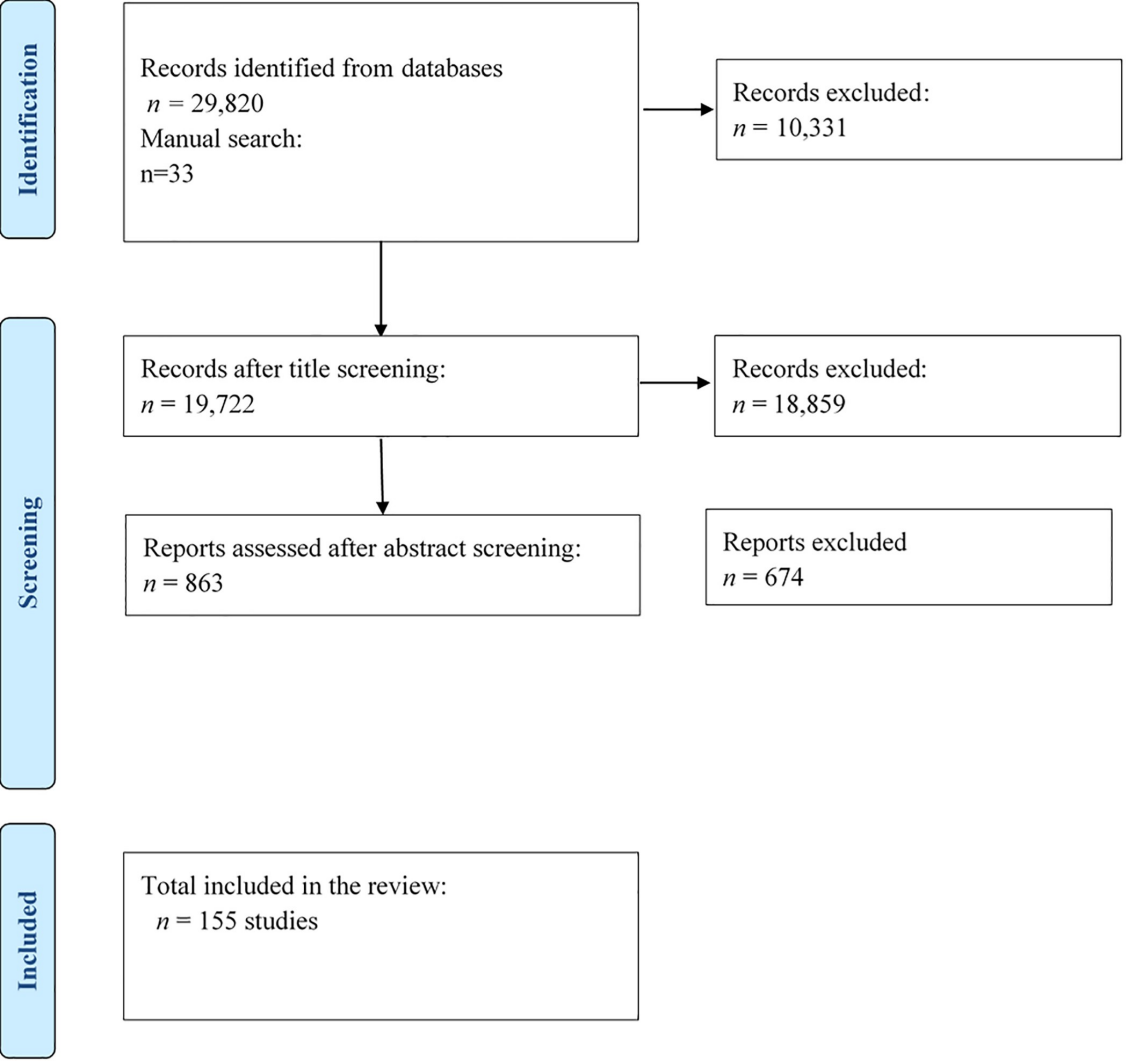
Prisma flow diagram of study selection.

The search followed the Preferred Reporting Items for Systematic reviews and Meta-Analyses (PRISMA) guidelines [10] [Fig 1]. A literature search in English and French in the following databases (PubMed, Cochrane, African Journals Online, CINAHL, African Index Medicus and Google Scholar) was conducted from December 2021 to February 2022 based on the predefined search strategy. Additional records were manually searched from reference lists of selected papers and grey literature. The search strategies used are attached in the supplementary appendix **S1 Table**.

### Article screening

Selection of peer-reviewed articles involved initial screening of title and abstracts to include studies that met the inclusion criteria. Then, full-text articles that fulfilled the inclusion criteria were retrieved, and screening was done. Data screening and extraction was independently conducted by three members of the research team, focusing on their areas of expertise: communicable diseases (CDs), non-communicable diseases (NCDs) and reproductive health, maternal neonatal, child and adolescent health (RMNCAH). The other two members of the review team with health economics background counter-checked the accuracy of screened and extracted information to ensure fidelity. Any disagreement was resolved through discussion and consensus-building. The articles that met the inclusion criteria were imported into EndNote X9. Duplicate records were identified and eliminated. Data from the included articles were extracted onto an Excel worksheet and the evidence graded. The outputs are presented according to the PRISMA (Preferred Reporting Items for Systematic Reviews and Meta-analysis) checklist in the supplementary appendix **S1 Checklist**.

### Data extraction

All relevant information required for analysis were collected using a data extraction template designed in Microsoft Excel 2013. Detailed data on the author, year of publication, the objective of the study, disease program/condition, population, country, study design, study year, intervention type, comparator, public health function, a measure of outcome (DALY), sample size and the summary findings from the study was conducted. Data extracted for included studies is in the supplementary appendix **S1 File.**

### Search results

The literature search yielded 29,853 articles, 29,820 titles and abstracts through database searching, and 33 records through bibliographic citation searches. A total of 19,722 articles were selected for the title and abstract evaluation. Full-text articles were then obtained for the 863 articles considered for inclusion, and then 151 full-text articles which met the inclusion criteria were included for the review. PRISMA flow [11] of selection and inclusion of the studies was used [Fig 1]. The list of the selected articles with their characteristics is provided as **S1 File**.

### Quality assessment

Quality assessment was conducted to appraise the quality of studies and establish the scientific processes involved in determining the value of high-quality research evidence to guide policy, inform decision making, direct resource allocation and leverage on strengths across similar contexts. The Quality of Health Economics Studies (QHES) 16-item appraisal tool, which is a validated quality-scoring instrument (with weight score range = 0–100; and >75 = high quality) [12] was used to garner the characteristics and quality of the included studies. Data extraction from the included studies was independently conducted by two research team members and compared for agreement; inter-rater agreement for the selection was 95%. Any disagreements were resolved through research team discussion, and agreement on scoring was determined at 79%. The quality indicators and their scoring are included in **S2 File.**

Based on the QHES appraisal, the minimum and maximum score of the included studies was 60 & 90, respectively, with an average quality score of 74 out of a possible 100. Almost 57% of studies obtained a fair good quality score of between 50–74%; and the remaining (43%) score was of high-quality score between 75–100%. None of the studies were extremely poor (0–24) or poor (25–49).

### Characteristics of included studies

Detailed data was extracted from included studies namely the author, year of publication, the objective of the study, disease program/condition, population, country, study design, study year, intervention type, comparator, public health function, measure of outcome, sample size, funding source declaration and the summary findings.

The majority of the effectiveness data was extracted from cost-effectiveness studies (80.8%) that used different types of modelling such as decision- analysis model, Markov’s model and micro-simulation methods among others while the remaining (19.2%) were built on other types of studies such as impact evaluations, economic evaluation among others. Different time horizons were used for modelling the data. A total of 35 (23.2%) decision model studies adopted annual time, 29 (19.2%) lifetime horizon and 27 (17.2%) used 10-year time horizon. For 16.6% of the studies, the period was not explicit or indicated. Most studies were carried out in the African region (43.5%) followed by Asia (20.1%) and multiple regions (12.3%). They included 75 (50.6%) articles for communicable diseases, 56 (36.4%) for NCDs and the remaining 19 (12.3%) for RMNCAH. Studies that covered interventions applicable to all age groups were the majority (24.1%) followed by reproductive age (15.1%) and early childhood (13.9%). Several studies covered the elderly in combination with adults while elderly specific studies were the least represented. Most interventions in the included studies addressed the public health function of disease prevention (41.2%), followed by treatment or curative care (28.6%) and health promotion (25.5%). A dearth of published literature on comparable effective interventions in the rehabilitation and palliative health functions existed. Table 1 below illustrates the characteristics of the included studies.

**Table 1:** Characteristics of included studies.

Characteristic	Percentage	Characteristic	Percentage
**Region**		**Publication years**	
Africa	43.5	2000–2009	18.5
Asia	20.1	2010–2019	64.3
Multi-regions	12.3	2020–2021	17.2
Americas	9.1	**Disease category**	
Europe	8.4	Communicable diseases	50.6
Australia	5.2	Non-Communicable diseases	36.4
Non-specific	1.3	RMNCAH	12.3
		CD/NCD	0.6
**Income level**		**Intervention type**	
LMIC	31.2	Curative	28.6
HIC	18.2	Disease prevention	41.2
LIC	13.0	Health promotion	25.5
Regional	11.0	Rehab/palliative	2.0
Global	9.1	Combination	2.8
UMIC	9.1	**Time horizon**	
Non-specific & combinations	8.4	Annual	23.2
**Study design**		Lifetime	19.2
Cost-effectiveness studies	80.8	10 years	17.2
Others*	19.2	Not indicated	16.6
		13–30 years	8.6
		2–7 years	8.6
		Other time horizons	6.6

*Economic evaluation, impact evaluations…

### Characterization of interventions

A total of 357 interventions that measured their effectiveness in DALYs were identified for the selected significant conditions per age cohort. At least 162 (45.74%) interventions for infectious diseases, 148 (41.5%) interventions for NCDs and the remaining 11.2% interventions for RMNCAH conditions across the age cohorts. The interventions were categorised first by the i) age cohort ii) disease area, and iii) public health function i.e., health promotion, disease prevention, curative, rehabilitative and palliative care. Almost half (53.5%) of the interventions were for five conditions (HIV, malaria, cancer, mental disorders and CVDs. Studies assessed the effectiveness of single interventions (58.2%), while others evaluated the effectiveness of multiple interventions (41.7%). It should be noted that these 357 interventions were not unique and some of them were repeated in several studies with different settings, comparators and coverages. For example, intermittent malaria preventive treatment in pregnant women (IPTp) was counted seven times as it appeared in five studies. However, only single and unique intervention was selected for the final menu. A detailed menu of effective interventions for each age cohort is attached in the supplementary appendix **S2 File**.

## Results

### Maternal disease burden and interventions

Pregnant mothers and newborns are considered a standard cohort due to their intertwined biological relationships, however, distinct health afflictions occur in these two populations. In the region, four maternal conditions dominate the disease burden related to pregnancy and delivery that can affect negatively the outcome of the mother and newborn. Maternal haemorrhage, maternal abortion and miscarriage, hypertensive disorders and maternal sepsis contribute immensely to the global burden of diseases but typically in the African region. At the end of 2019, maternal disorders together contributed 11,533,751.36 DALYs in the African region. Forty five percent of the DALYs due to maternal conditions were contributed by the African region while almost 70% of the DALYs caused by maternal abortions and 60% of maternal sepsis were contributed by the region. These five maternal conditions were responsible for 32.3% of the total maternal disorder DALYs in the region and 46% of the global ones [Table 2]. This makes them important public health problems for countries to address them in their pursuit to reduce the maternal and neonatal mortalities.

**Table 2:** Leading maternal conditions in the Africa region, global and African region DALYs contribution, 2019.

Rank	Maternal disorders	DALYs, Africa region	%	DALYs, Global	Africa contribution (%)
1	Maternal haemorrhage	1,094,474.07	9.5	3,087,856.85	35.4
2	Maternal abortion and miscarriage	783,235.92	6.8	1,130,038.21	69.3
3	Maternal hypertensive disorders	712,796.11	6.2	1,823,862.91	39.1
4	Maternal sepsis and other maternal infections	633,631.10	5.5	1,064,670.02	59.5
5	Maternal obstructed labour and uterine rupture	504,551.99	4.4	999,540.67	50.5
	**Subtotal**	**3,728,689.18**	32.3	**8,105,968.66**	**46.0**
	**Total for all maternal disorders**	**11,533,751.36**	100.0	**25,352,807.46**	**45.5**

Overall, ten (n = 10) studies were identified with interventions that can reduce the leading causes of DALYs. Health promotion (n = 2); Disease Prevention (n = 4); Curative (n = 4); Rehabilitative/Palliative Care (n = 0)

#### Health promotion

Two (2) health promotion studies were identified; these included for obstetric haemorrhage [13]; and hypertensive disorders of pregnancy [14]. A quality improvement programme covering personnel, system management and quality communication at facility levels was found to be effective at averting DALYs associated with obstetric haemorrhage [13]. Research evidence found that promoting the use of simple diagnostic tools in urine assessment was critical for identifying early signs of preeclampsia and was effective at averting DALYs associated with hypertensive disorders of pregnancy [14].

#### Disease prevention

We identified four (n = 4) studies; one on obstetric haemorrhage [15]; two (n = 2) on unsafe abortions [13, 15] and one on maternal sepsis [15]. In the management of obstetric haemorrhage, early application of Non-pneumatic Anti-Shock Garment (NASG) at the primary health care level for women in hypovolemic shock can be -effective across diverse clinical settings rather than its application at the regional level [16]. The NASG is a low-cost first-aid compression device that limits the effects of PPH. Concerning unsafe abortions and its complications, sustained provision of Long-acting reversible contraceptives (LARCs) and depot-medroxyprogesterone acetate [DMPA] methods were effective in averting DALYs associated with unsafe abortions and its-related complications [17]. Women who were well counselled used these methods for a long time culminating into maternal and child health benefits. Vaginal misoprostol was found to be effective against the effects of unsafe abortions [15]. Tetanus toxoid for pregnant women significantly for averted DALYs associated with maternal and neonatal conditions; therefore, investing in universal coverage of this intervention and its scale-up was recommended [15].

#### Curative

We identified (n = 3) studies covering diverse interventions for obstetric haemorrhage (n = 3); and hypertensive disorders in pregnancy (n = 1). Concerning obstetric haemorrhage, Every Second Matters—Uterine Balloon Tamponade (ESM-UBT) was found to be effective and less expensive for managing atonic postpartum haemorrhage and averting DALYs compared to standard care—condom uterine balloon tamponade [18]. In another study, the use of NASG across three intervention scenarios: no women in shock receive the NASG, only women in severe shock receive the NASG, and all women in shock receive the NASG to be effective [19]. The application of NASG to women with haemorrhage significantly affected averting DALYs and decreasing mortality. Active management of the third stage of labour—prophylactic oxytocin, cord clamping and delivery of the placenta by controlled cord traction was effective for averting DALYs associated with obstetric haemorrhage [15]. In relation to preeclampsia and eclampsia management, packages of care involving anti-hypertensives and magnesium sulphates effectively averted DALYs associated with hypertensive disorders in pregnancy, hence, calling for universal coverage of the intervention and its scale-up [15]. In maternal sepsis case management, inpatient care, including treatment with antibiotics, were effective.

### Disease burden and interventions in newborn/neonate cohort

Newborns, also known as neonates, are considered individuals aged from birth up to 28 days of life. Complications from birth asphyxia and birth trauma followed by preterm birth and neonatal sepsis dominate the disease burden. These are followed by lower respiratory infections, congenital anomalies and syphilis. At the end of 2019, three of the leading conditions and diseases related to complicated birth contributed a total of 55,801, 796.50 DALYs, accounting for 62.2% of the DALYs in the African region and 25.8% of the Global DALYs in the neonate age group [Table 3].

**Table 3:** Leading conditions in neonate age cohort in the Africa region, global and African region DALYs contribution, 2019.

Rank	Conditions	DALYs, Africa Region	%	DALYs, Global	African contribution (%)
1	Neonatal encephalopathy due to birth asphyxia and trauma	24,135,392.07	26.9	48,982,146.29	49.3
2	Neonatal preterm birth	21,544,682.64	24.0	56,492,629.93	38.1
3	Neonatal sepsis and other neonatal infections	10,121,721.79	11.3	18,688,688.55	54.2
4	Lower respiratory infections	7,762,683.22	8.7	17,442,098.14	44.5
5	Congenital abnormalities*	5,375,147.82	6.0	16,467,535.72	32.6
6	Syphilis	3,542,416.85	3.9	5,475,407.22	64.7
7	Diarrheal diseases	2,124,956.32	2.4	3,878,981.52	54.8
	***Subtotal***	***74*,*607*,*000*.*72***	***83*.*2***	***167*,*427*,*487*.*36***	**44.6**
	***Total DALYs*, *Neonates***	***89*,*700*,*472*.*10***	***100*.*0***	***215*,*978*,*571*.*79***	**41.5**

*Congenital abnormalities include congenital heart anomalies, congenital physical and limb defects, neural tube defect and other congenital birth defects.

Overall, seven (n = 7) studies with interventions that can reduce the leading causes of DALYs were identified. Health promotion (n = 4); Disease Prevention (n = 2); Curative (n = 1); Rehabilitative/Palliative Care (n = 0).

#### Health promotion

One study (n = 1) reported on health promotion campaigns for supplemental folate/folic acid consumption first, in women capable of or planning a pregnancy and second in population-wide campaigns to promote supplement use where mandatory fortification was found to be the most effective intervention at reducing neural tube defects [20]. The other study (n = 1) reported on screening all newborn using three possible screening options: pulse oximetry alone, clinical assessment alone, and pulse as an adjunct to clinical assessment in diagnosing critical congenital heart disease [21]. The most effective of the three neonatal screening options was pulse oximetry with clinical assessment. Newborn screening at the national level was recommended as an effective measure for early diagnosis and subsequent intervention to expand long term national health benefits and improve newborn health outcomes. These interventions were recommended across all levels of care, i.e., community, primary, secondary and tertiary. Ali et al., [22] reported on the use of the Augmented Infant Resuscitator (AIR) device as an effective device in a mixed microsimulation calibrated Markov model of the apnoea training programme when used as a supplement to existing resuscitation training programs. More DALYs were estimated to be averted on top of the benefits of the widely adopted Helping Babies Breathe (HBB) training for improving the quality of bag-valve-mask resuscitation among non-breathing newborn. The benefits of this AIR device have been identified; however, prioritisation of HBB implementation in birthing facilities was recommended until field test confirmations of the AIR device are conducted at scale. For intrapartum related complications, we identified that low-dose, high-frequency training in basic emergency obstetrics and newborn was an effective strategy in improving newborn health outcomes and averting DALYs. Integrating this intervention into existing in-service training programs and health systems is important for achieving quality health care outcomes [23].

#### Disease prevention

Two (n = 2) studies were identified [15, 24], one related to neonatal sepsis and the other, on preterm birth complications. Research evidence from Ahmed et al., found that Group B streptococcal (GBS) hexavalent maternal vaccination program would avert more DALYs than the current standard of care for preventing neonatal sepsis [24]. The other study found that administration of- antibiotics at institutional levels for preterm pre-labour rupture of membrane (pPRoM), antenatal corticosteroids for preterm labour and Kangaroo mother care effectively averted DALYs associated with preterm birth complications in study [15].

#### Curative

Injectable antibiotics were found to be effective for averting DALYs [15]. For infants with respiratory distress at birth identified as an intra-partum-related complication, immediate neonatal resuscitation was effective in averting both short and long sequelae of hypoxic-ischemic encephalopathy [15].

### Disease burden and interventions in early childhood (under 5 years)

The major contributors to DALYs for this age cohort were communicable diseases such as diarrhoeal diseases, lower respiratory infections, and malaria. Sequel of births such neonatal encephalopathy due to birth asphyxia and trauma, preterm birth and sepsis were also important contributors of DALY. This could be as a result of overlap with that of newborns. Three of the leading causes of DALYs in the under 5 age group in the African region (diarrheal diseases, lower respiratory tract infections and malaria) contributed a total of 91,744,812.03 DALYs which is 38.3% for the African region and 19.3% of the global DALYs for these three conditions in this age group. Almost 96% of global malaria, 68.3% of meningitis and 67.9% of the diarrhoea DALYs in this age group were contributed by the African region [Table 4].

**Table 4:** Leading conditions in under-five year age cohort in the Africa region, global and African region DALYs contribution, 2019.

Rank	Conditions	DALYs, African Region	%	DALYs, Global	Africa Contribution (%)
1	Diarrheal diseases	30,925,537.71	12.9	45,544,641.05	**67.9**
2	Lower respiratory infections	30,503,932.72	12.7	59,162,236.12	**51.6**
3	Malaria	30,315,341.60	12.6	31,594,030.51	**96.0**
4	Neonatal encephalopathy due to birth asphyxia and trauma	24,653,499.91	10.3	50,775,729.16	**48.6**
5	Neonatal preterm birth	22,356,728.45	9.3	60,200,062.02	**37.1**
6	Neonatal sepsis and other neonatal infections	10,735,404.53	4.5	20,573,473.61	**52.2**
7	Meningitis	6,743,317.42	2.8	9,876,050.87	**68.3**
	***Subtotal***	***156*,*233*,*762*.*35***	***65*.*2***	***277*,*726*,*223*.*34***	**56.3**
	***Total DALYs*, *Under-five***	***239*,*666*,*918*.*68***	***100*.*0***	***475*,*247*,*148*.*09***	**50.4**

High impact interventions that reduce these leading causes of DALYs in this age group are described below.

Overall, fifty-five (n = 55) studies were identified. Health promotion (n = 14); Disease Prevention (n = 28); Curative (n = 13); Rehabilitative/Palliative Care (n = 0)

#### Health promotion

We found several effective health promotion interventions for children aged 0–5. Exclusive breastfeeding promotion was highlighted in several studies as an effective intervention in averting DALYs for; diarrhoeal diseases [25, 26] and LRTIs [27]. No significant difference was found in community-based peer counselling to promote exclusive breastfeeding (in addition to HFP) relative to standard HFP (Health Facility Breastfeeding promotion) [26]. The promotion of population-wide oral rehydration therapy (ORT) was also found to be effective in averting DALYs caused by diarrhoeal diseases for this age cohort [25].

Community-based information activities was an effective strategy to avert DALYs caused by malaria [28]. Promotion of nutrient-rich diets through price subsidies, tax incentives and disincentives, and cash incentives effectively averted DALYs caused by malnutrition. Examples of effective price subsidies strategies included; price subsidies for fortified packaged infant cereals (F-PICs) for under 2’s within poorer households [29], price subsidies on fortified packaged complementary foods (FPCF) for children in different wealth quintiles [30]. Cash incentives such as standard cashback (SCB), double cash (DCB), and fresh food vouchers (FFV) also had a significant impact on nutrition outcomes in children under five years of age [31]. Other nutrition-based incentives included the mass distribution of daily micronutrient powders (MNPs) for infants between 6–18 months [32] and routine supplementation with EMMS [33]. Mass media campaigns addressing the main causes of post neonatal child mortality such as malnutrition were effective strategies in averting DALYs for this age cohort [34, 35]. All these interventions were implemented at the community level.

#### Disease prevention

Effective disease prevention interventions covering the major causes of DALYs for this age cohort included; vaccinations for various diseases and conditions such as Pneumococcal vaccine for pneumonia [27, 36–38], Haemophilus influenzae type B vaccination [27], cholera vaccination [39], RTS,S malaria vaccine [40, 41] and vaccinations for meningitis [42, 43]. Combinations of interventions such as pneumococcal conjugate vaccine and Haemophilus influenza type B vaccine averted more DALYs [27] than single entity vaccinations.

Exclusive breastfeeding for the first 6 months of life was an effective disease prevention strategy in averting DALYs for malnutrition [44]. Examples of nutrition modifications to prevent disease included; daily iron sulphate II supplementation [45], complementary feeding practices in infants six months to 3 years of age [44] and zinc supplementation for LTRIs [30] and diarrhoeal diseases [46].

Several interventions were effective in averting DALYs caused by malaria through disease prevention. These interventions included; intermittent preventive treatment of malaria in infants (IPTi) [47], intermittent malaria screening of school children [48], intermittent preventive treatment of malaria in pregnancy (IPTp) [49, 50], seasonal malaria chemoprophylaxis (SMC) [51], insecticide-treated nets (ITNs) [48, 49, 52, 53], indoor residual spraying (IRS) [25, 48, 49, 53], larviciding [54]. But WHO guidelines recommend larviciding as supplementary intervention in areas where optimal coverage with ITNs or IRS has been achieved., Long lasting ITNs averted more DALYs compared to conventional ITNs. Combinations of interventions averted more DALYs for example, IPTp + ITN [50], IRS with pirimiphos-methyl and pyrethroid ITNs [49, 53, 55], LIN plus IRS [48]. However, WHO guidelines recommend against combining ITNs and IRS and that priority be given to delivering either ITNs or IRS at optimal coverage and to a high standard.

Inhaled pollutants were considered risk factors for both infectious and non-infectious respiratory diseases; hence, the use of solid stoves and cleaner liquid fuels effectively averted DALYs caused by inhalation of polluted air [27, 56].

#### Curative

Effective curative interventions for LRTi included; treatment of non-severe clinical pneumonia at the community level and facility level [25, 27, 46, 57], zinc supplementation [27], standard of care (hospitalization, low flow oxygen and antibiotics) and bubble continuous positive airway (bCPAP) [58]. Diarrhoeal disease curative interventions included; oral rehydration therapy [46], therapeutic zinc supplementation [46], case management [46], and complementary feeding [44]. Malaria curative interventions included case management with artemisinin-based combination therapy [49], prereferral rectal artesunate for treatment of severe malaria [59] and parenteral artesunate for treating severe malaria [60].

### Disease burden and interventions in late childhood (5–9 years)

The age group 5–9 (taken as a proxy for the 6–11 years old cohort), contributes the least DALYs, 3.4% for the African region and 2% globally among the six age cohorts. The major DALY contributors in this age group in 2019 were malaria, dietary iron deficiency, diarrheal diseases, and HIV. Lower respiratory tract infections, Invasive Non-typhoidal Salmonella (iNTS) and meningitis were also leading contributors of DALYs in this age group. In this age group, 80.1% of malaria, 86.7% of HIV/AIDS and 83.5% of Invasive Non-typhoidal Salmonella (iNTS) of the global DALYs were contributed by the African region [Table 5]. Due to the lack of data that measures effective interventions using DALYs averted as the outcome measure, invasive Non-typhoidal Salmonella is not addressed here.

**Table 5:** Leading conditions in 5–9 age cohort in the Africa region, global and African region DALYs contribution, 2019.

Rank	Conditions	DALYs, African Region	% DALYs	DALYs, Global	Africa Contribution (%)
1	Malaria	2,088,923.73	9.8	2,607,727.98	**80.1**
2	Dietary iron deficiency	2,026,596.59	9.5	5,432,574.74	**37.3**
3	Diarrheal diseases	1,707,585.99	8.0	3,898,530.44	**43.8**
4	HIV/AIDS	938,149.16	4.4	1,081,973.66	**86.7**
5	Lower respiratory infections	904,461.15	4.3	2,297,525.32	**39.4**
6	Invasive Non-typhoidal Salmonella (iNTS)	673,233.82	3.2	806,327.59	**83.5**
7	Meningitis	652,749.20	3.1	1,172,487.85	**55.7**
	***Subtotal***	***8*,*991*,*699*.*63***	*42*.*3*	***17*,*297*,*147*.*58***	**52.0**
	***Total DALYs*, *5–9 years***	***21*,*263*,*276*.*10***	*100*.*0*	***62*,*227*,*042*.*60***	**34.2**

Overall, forty-four (n = 44) studies were identified to address these leading conditions. Health promotion (n = 16); Disease Prevention (n = 15); Curative (n = 13); Rehabilitative/Palliative Care (n = 0)

#### Health promotion

Malaria, dietary iron deficiency, diarrhoeal and meningitis health promotion interventions for this age cohort were similar to those in the early childhood group, with exceptions such as breastfeeding promotion for diarrhoeal diseases. Promotion of nutrient-rich diets through price subsidies, tax incentives and disincentives, and cash incentives effectively avert DALYs caused by malnutrition. These have been discussed within the early childhood section [29–35, 61]. Population-wide ORT interventions were important for diarrhoeal diseases [25].

We did not find any health promotion interventions for LRTIs for this age cohort. Meningitis health promotion interventions have also been addressed in the early childhood section [62]. HIV based interventions such as school-based sexual education on HIV and mass media campaigns were significant in averting DALYs related to HIV [63, 64]. Although at higher budget levels, school-based education yielded higher health gains than mass media, combining the two strategies averted the most DALYs caused by HIV. Information education and Communication (IEC) programmes also effectively averted DALYs in HIV disease [65]. Home-based HIV testing for partners of pregnant women was found to be effective [65].

#### Disease prevention

Malaria, dietary iron deficiency, diarrhoeal diseases, LRTIs and meningitis disease prevention interventions for this age cohort were similar to those in the early childhood group, with exceptions such as exclusive breastfeeding and complementary feeding practices unique to the younger age cohorts. These have been addressed within the early childhood section. Effective disease prevention interventions for HIV/AIDS included; blood safety (63,64), voluntary counselling and testing [62, 66], street children programs [64], treatment for STIs [62, 64, 67] and prevention of mother to child transmission [62, 64].

#### Curative

Curative interventions for this age cohort were similar to those of early childhood for malaria, malnutrition, diarrhoeal diseases, LRTIs and meningitis except for exclusive breastfeeding and complementary feeding practices for malnutrition. These have been discussed within the early childhood interventions section. HIV based curative interventions that were found to be effective in averting more DALYs included; highly active antiretroviral therapy (HAART) and HAART with laboratory monitoring i.e., CD4 and viral load [68].

### Disease burden and interventions in Adolescents (12–24 years)

In the age group 10–24 (taken as a proxy for the 12–24 years old cohort), the major DALY contributors were HIV/AIDS, malaria, diarrheal diseases and TB. Three non-communicable diseases such as migraine, road injuries and major depressive disorders appeared as leading conditions in this age group. In 2019, this age group was responsible for 9.2% of the total DALYs in the African region and 7.8% of the global DALYs. In this age group, HIV/AIDS and malaria in the African region contributed 83.2% and 81.0%, respectively of the Global DALYs for these diseases [Table 6].

**Table 6:** Leading conditions in 10–24 age cohort in the Africa region, global and African region DALY contribution, 2019.

Rank	Conditions	DALY, African Region	%	DALYs, Global	Africa Contribution (%)
1	HIV/AIDS	4,901,169.97	8.6	5,892,211.31	**83.2**
2	Malaria	3,351,548.39	5.9	4,137,889.76	**81.0**
3	Diarrheal diseases	2,142,777.42	3.8	5,888,885.30	**36.4**
4	Drug-susceptible tuberculosis	1,847,994.64	3.3	4,441,577.08	**41.6**
5	Migraine	1,752,739.90	3.1	10,776,503.35	**16.3**
6	Motor vehicle road injuries	1,615,664.15	2.8	6,022,887.08	**26.8**
7	Major depressive disorder	1,511,328.66	2.7	7,170,287.25	**21.1**
	***Subtotal***	***17*,*123*,*223*.*12***	***30*.*2***	***44*,*330*,*241*.*12***	**38.6**
	***Total DALY*, *10–24 Years***	***56*,*755*,*881*.*80***	***100*.*0***	***237*,*718*,*695*.*90***	**23.9**

Overall, fifty-seven (n = 57) studies were identified that address the leading conditions. Health promotion (n = 11); Disease Prevention (n = 24); Curative (n = 22); Rehabilitative/Palliative Care (n = 0)

#### Health promotion

HIV/AIDS health promotion interventions were similar to those of the late childhood age cohort and have been addressed in that section with the addition of condom promotion. Malaria disease health promotion interventions that were effective in averting DALYs have been addressed within the early childhood section.

Effective health promotion interventions that covered road traffic injuries and mental conditions included; increased taxation on alcohol, increased minimum legal drinking age, alcohol advertising bans and limited hours of alcohol sale [69, 70]. Random breath testing, selective breath testing, and mass media campaigns on road safety were also found to be effective against road traffic injuries [71]. Additionally, taxation of sugar-sweetened beverages to prevent obesity was found to be effective in averting DALYs caused by mental health conditions [35, 72].

#### Disease prevention

HIV/AIDS disease prevention interventions were similar to the late childhood age cohort. However, there were additional HIV/AIDS interventions for this age cohort and these included; Voluntary medical male circumcision [73], provision of oral PrEP ([74], intravaginal rings [74], microbicide gels, SILCS diaphragms used in concert with gel [74], injectable PrEP, provision of dual PrEP (both oral and injectable PrEP) and condom use [64, 65, 75, 76]. Malaria disease prevention interventions such ITNs, IRS, larviciding were effective in averting DALYs caused by malaria. These have been addressed within the early childhood cohort. Short course TB preventive therapy effectively averted TB DALYs [77].

Enforcement of road safety regulations through various means was found to be effective in averting DALYs. These included; speed limits via mobile speed cameras, breath testing campaigns, seatbelt use in cars, helmet use by motorcyclists and bicyclists, traffic codes, and building speed bumps at high-risk intersections [69, 70, 78]. School-based psychological interventions to prevent the onset of depression among students by a universal or targeted approach was an effective preventive method of averting DALYs caused by mental conditions [79].

#### Curative

Effective curative interventions for HIV/AIDS were similar to those of late childhood and are addressed in that section. Effective curative interventions for diarrheal diseases were similar to those addressed within the early childhood section. TB effective curative interventions included; Diagnosis using Xpert MTB/RIF [61, 80], DOTS for management of TB [62, 81], DOTS-Plus treatment for multidrug-resistant tuberculosis [82], Active case finding (ACF) for TB in household contacts of index smear-positive TB patients [83]. Increased access to surgery significantly averted DALYs for road traffic injury victims. Mental conditions’ effective interventions included; community-based pharmacologic and psychosocial treatment for depression [75, 84, 85], roadside breath testing for alcohol use disorder [75], increased access to newer antipsychotic medications and psychosocial treatment [75, 84, 85], increased access to older mood stabilizers medications, newer antidepressant and psychosocial treatment for bipolar disorder [85], integration of family intervention with antipsychotic medications for schizophrenia, eHealth sessions [86] and face-to-face sessions [86, 87].

### Disease burden and interventions in adults (25–59 years)

In Africa, this age group is ravaged by diseases of both infectious and non-communicable nature. HIV/AIDS is the leading condition, with almost 17% of DALYs in this cohort attributed to this single disease. This same population suffers from cardiovascular diseases, diabetes mellitus type 2 and cancers, which contribute 13.6% of the total DALYs in the African region. Almost one out of five (24%) of the DALYs in the African region are contributed by this age group. In this age group, two communicable diseases such as HIV/AIDS and malaria contributed 72% and 87% of the global DALYs, while cardiovascular diseases, diabetes mellitus and total cancers contributed only 16.6% of the global DALYs [Table 7]. This indicates that even in the adult population of the African region HIV/AIDS, tuberculosis and malaria remain significant public health problems despite the increasing NCD trends.

**Table 7:** Leading conditions in 25–59 age cohort in the Africa region, global and African region DALYs contribution, 2019.

Rank	Conditions	DALYs, African Region	% DALYs	DALYs, Global	Africa Contribution (%)
1	HIV/AIDS	24,801,031.79	16.7	34,446,312.72	**72.0**
2	CVD*+DM	12,068,261.73	8.1	139,514,387.87	**8.7**
3	Drug-susceptible tuberculosis	8,201,866.33	5.5	23,401,190.59	**35.0**
4	Total cancers	8,121,182.55	5.5	102,362,972.15	**7.9**
5	Malaria	5,220,940.27	3.5	5,997,964.72	**87.0**
6	Diarrheal diseases	3,942,411.44	2.6	11,767,585.64	**33.5**
7	Major depressive disorder	3,135,647.97	2.1	22,389,053.94	**14.0**
	***Subtotal***	***65*,*491*,*342*.*08***	***44*.*0***	***339*,*879*,*467*.*63***	**19.3**
	**Total DALYS in 25–59 Years**	**148,786,945.64**		**1,069,431,992.99**	

* CVD combines the following conditions: ischemic heart disease, intracerebral haemorrhage, ischemic stroke, hypertensive heart disease, rheumatic heart diseases, congenital heart diseases and peripheral artery disease. Cardiovascular diseases and diabetes mellitus are considered together as the interventions that address the risk factors are similar.

Overall, seventy-eight (n = 78) studies were identified that address the leading conditions in this age group. Health promotion (n = 16); Disease Prevention (n = 44); Curative (n = 17); Rehabilitative/Palliative Care (n = 1).

#### Health promotion

In addition to earlier highlighted HIV based health promotion interventions, including school-based sexual education on HIV and mass media campaigns [62, 63], peer-led outreach and education [63, 67], peer education for sex workers [62], condom promotion for female sex workers (FSWs) and men who have sex with men [68] and condom distribution and promotion for sexually active populations were effective in averting HIV DALYs for this age cohort [63, 67]. Community-based information activities on malaria were found to be effective in averting DALYs caused by malaria [27]. Diet-related interventions had a significant role in averting DALYs for CVD/DM and cancers. These interventions included legislation over the following; population-wide reduction in daily salt consumption [54, 88, 89], tax reduction on fruits and vegetables, tax increase on fats and sugar, taxation of junk food and front of pack traffic light labelling of nutrition on processed foods [34]. Cancer awareness interventions averted cancer DALYs for this age cohort [90]. A combined approach to the prevention of behavioural risk factors was found to avert DALYs caused by NCDs significantly.

#### Disease prevention

Sustained free HIV voluntary counselling and testing [62, 64, 66], home-based HIV testing of pregnant womens’ [65], peer counselling for key populations [62, 63, 67], treatment of sexually transmitted infections (STIs) for the general population [67] and for key populations [62, 63], oral pre-exposure prophylaxis (Vogelzang et al. 2020, [28, 73, 74]), oral pre-exposure prophylaxis (PrEP) for MSMs [91], voluntary male medical circumcision [72], PMTCT [65] were found to be effective HIV disease prevention interventions and antenatal syphilis screening was effective in prevention syphilis in sub-Saharan Africa [92]. Dual-use of womens’ condoms for family planning and HIV prevention were shown to avert DALYs due to HIV [76]. The additional interventions for this age cohort mostly comprise interventions for key populations. TB preventive treatment for persons living with HIV [77] and tuberculin skin testing (TST) followed by IPT for TST-positive patients with no evidence of active TB was found to avert DALYs caused by TB [93] significantly. Malaria prevention for this age cohort also benefited significantly from interventions such as ITNs, IRS, IRS and ITN and larviciding, which have been addressed in earlier sections.

Cancer preventive interventions that were effective for this age cohort included; HPV cytology screening/triennial pap smear [94, 95] and HPV vaccination in pre-adolescent girls for cervical cancer [94, 96], biennial mammography in women above 40 years [90, 95, 97, 98] and biennial clinical breast examination for breast cancer [90, 97], colonoscopy every five years for colon cancer [95] and genomic screening of individuals at risk [53]. The effectiveness of annual mammography was found to be low due to the short interval of screening (Zehtab et al. [98]). Colonoscopy screening every seven years was the most effective strategy in terms of DALYs averted. However, it carried a high number of iatrogenic deaths associated with endoscopy-related perforations, while colonoscopy screening every five years was found to generate the most significant number of iatrogenic deaths [95]. Combined HPV vaccination in pre-adolescence, followed by screening (using HPV DNA testing) in adulthood, was also highly effective compared to one intervention [94].

Interventions for CVD/DM prevention that were effective included; community-based management of hypertension targeting everyone regardless of hypertension status as opposed to targeting only people with hypertension [99, 100], screening and addressing CVD risk factors [101–103], E-health interventions for CVD risk assessment and mitigation [103], integration of hypertension and diabetes screening with HIV programs [104] and use of polypill for secondary CVD prevention in high-risk candidates [25]. Reducing complications from diabetes in LMICs through scaling up blood pressure and statin medication treatment initiation and blood pressure medication titration was found to be a more effective strategy rather than focusing on increasing screening to increase diabetes diagnosis or a glycaemic treatment and control among people with diabetes [101].

#### Curative

Highly active antiretroviral therapy with and without laboratory monitoring [62, 63, 68], injectable cabotegravir/rilpivirine in all people on ART [105] and co-morbidity and opportunistic infections treatment were effective curative interventions for HIV/AIDS. Lateral-flow urine lipoarabinomannan (LAM) with a standard for TB diagnosis in PLHIV [106], active case finding (ACF) for TB in household contacts of index smear-positive TB patients with Xpert MTB/RIF as a diagnostic tool [83], Xpert/LF-LAM diagnosis [107], DOTS for management of TB [25, 73, 81, 82, 108]. DOTS management of TB was family-based, community-based or facility-based. DOTS plus using Bedaquiline based regimen was also effective in MDR TB cure [109]. Case management with artemisinin-based combination therapy with high coverage [48] and parenteral artesunate for treating severe malaria [59] were effective in Malaria cure.

Improved access to cancer treatment averted cancer DALYs significantly [90]. Nicotine replacement and metformin for diabetes treatment were effective for CVD/DM [25].

#### Rehabilitative and palliative care

Basic palliative care (palliative care volunteers training programme + home-based visits by volunteers every fortnight + pain treatment through morphine, laxatives and palliative radiotherapy for eligible patients) and extended that replaces the volunteers with nurses and strengthened pain management with antidepressants, anti-emetics and zelodronic acid averted breast cancer DALYs in Ghana [90].

### Disease burden and interventions in Elderly (Above 60 years)

The leading causes of DALYs in this age group (60+ years) were the non-communicable diseases such as cardiovascular diseases (CVDs), cancers and type 2 diabetes mellitus. Almost 35% of the DALYs for this age group in the African region were contributed by cardiovascular diseases, diabetes mellitus and cancers. Another non-communicable disease with significant contributions to the DALYs in the region was chronic obstructive pulmonary diseases. The dominance of non-communicable diseases in this cohort demonstrates well the effects of epidemiological transition that Africa is experiencing and the opportunities to avert future burden by applying effective interventions in the younger populations. In 2019, leading non-communicable conditions such as CVD with diabetes mellitus, total cancers and COPD in the African region contributed 6.1%, 4.4% and 3.3% of the global DALYs, respectively, while almost 90.8% of the global malaria burden for this age group was contributed by the African region [Table 8].

**Table 8:** Leading conditions in 60+ age cohort in the Africa region, global and African region DALYs contribution, 2019.

Rank	60+ Conditions	DALYs, African Region	% DALYs	DALYs, Global	Africa Contribution (%)
1	CVD+DM	16,411,642.68	26.1	268,943,113.46	**6.1**
2	Total cancers	5,864,268.24	9.3	134,113,016.07	**4.4**
3	Lower respiratory infections	3,389,037.25	5.4	20,745,385.95	**16.3**
4	Drug-susceptible tuberculosis	2,339,599.19	3.7	9,730,629.95	**24.0**
5	Diarrheal diseases	2,199,841.57	3.5	13,818,136.55	**15.9**
6	Malaria	1,906,745.18	3.0	2,100,198.19	**90.8**
7	Chronic obstructive pulmonary disease	1,904,720.00	3.0	57,582,989.97	**3.3**
	***Subtotal***	***34*,*015*,*854*.*11***	***54*.*2***	***507*,*033*,*470*.*13***	**6.7**
	***Total DALY*, *60+ years***	***62*,*772*,*598*.*30***	***100*.*0***	***981*,*472*,*243*.*67***	**6.4**

Overall, sixty-two (n = 62) studies were identified. Health promotion (n = 9); Disease Prevention (n = 33); Curative (n = 16); Rehabilitative/Palliative Care (n = 4).

#### Health promotion

Diet based health promotion interventions that averted DALYs for NCDs, including CVD/DM and cancers, were; mass media campaigns on salt reduction [110], government legislation to reduce salt consumption [88], taxation of junk food, fats and sugars [111], front of pack-traffic light labelling of nutrition of processed foods [34], lower consumption of meat, dairy and fats [112] and reduction of tax for fruits and vegetables [111]. Cancer awareness-raising was effective in averting DALYs for cancers [90], and a combined approach in the prevention of behavioural risk factors was found to be very effective for NCDs generally. Effective interventions aimed at reducing the harmful effects of tobacco included tobacco price increase [25] and media campaigns on smoking [110].

Replacement of traditional stoves with solid fuel or liquid gas and attainment of air quality PM 2.5 concentration significantly was found to translate into an impressively large health gain [56, 113]. Among all these health promotion interventions, five interventions were based on LMIC settings in two countries (Vietnam and India). These included; mass media campaigns on salt reduction (Vietnam), front of pack-traffic light labelling of nutrition of processed foods (India), increased taxation on junk foods (India), legislations to replace traditional stoves with solid fuel or liquid gas (India) and attainment of air quality PM 2.5 concentration (India). The effect of legislation on salt reduction consumption was evaluated across multiple countries of all income classifications, while the rest of the interventions were based on high-income settings.

#### Disease prevention

Interventions to prevent CVD/DM that were effectively included; community-based management of hypertension [25, 99], screening and addressing CVD risk factors [100, 114], mobile technology/E-health enabled community-based CVD risk assessment mechanisms [103], higher consumption of grain-based foods, seafoods, fruits and vegetables [60, 102, 111, 112] and lower consumption of meat, dairy and fats [112]. Cancer disease prevention interventions that were effective were similar to that of the adult cohort addressed above.

LRTI interventions that were found to be effective included; COVID Vaccine) [115], H Influenzae type B vaccination [18], use of solid stoves [26, 56], use of cleaner liquid fuels [26, 56]. Replacement of indoor stoves with smokeless technology was effective in averting DALYs for COPD [56].

#### Curative

Improved access to breast cancer treatment averted cancer DALYs [90]. Availability of metformin for diabetes treatment prevented CVD/DM DALYS [25]. Effective LRTI curative interventions included; treatment of non-severe clinical pneumonia at the community level [25, 26, 45], treatment of non-severe and severe clinical pneumonia at the facility level [26], the standard of care (hospitalization, low flow oxygen and antibiotics [26]. Improved access to inhaled corticosteroids and long-acting β agonists for asthma and COPD was found to be effective [116].

#### Rehabilitative and palliative care

Basic palliative care (palliative care volunteers training programme + home-based visits by volunteers every fortnight + pain treatment through morphine, laxatives and palliative radiotherapy for eligible patients) and extended that replaces the volunteers with nurses and strengthened pain management with antidepressants, anti-emetics and zelodronic acid averted breast cancer DALYs in Ghana [90].

Using QALY as an outcome measure for rehabilitative and palliative care interventions, we found interventions for the adult and elderly groups and the control of cardiovascular diseases and COPD. In combating cardiovascular disease, including diabetes among adults, home-based cardiac rehabilitation was an effective intervention than usual care alone in patients with reduced ejection fraction heart failure [117]. Similarly, person-centred care services delivered at the primary or secondary level, when added to usual care in chronic heart failure or COPD for individuals, can improve patients’ quality of life [118]. Among the elderly, in addition to the interventions listed above, in the palliative care setting, two additional interventions that resulted in QALY gains were person-centred home-based palliative care and home-based care for advanced heart failure by medical personnel [119, 120].

## Discussion

This comprehensive review has documented the leading causes of DALYs and identified effective interventions for the leading conditions in the African region. Based on this analysis, a menu of effective interventions that could avert most of the DALYs for the major diseases and conditions were identified by age cohort, public health functions and levels of care.

Most of these interventions are almost compatible with those generated by UHC compendium database [121], WHO AFRO essential health interventions [122], WHO-CHOICE [2] and DCP3 [123]. This is aligned to the ongoing regional shift, placing emphasis on the continuum of care across the public health function and throughout the life course. The structural alignment also builds on the foundation that interventions are most effective when targeted to the population of interest. In this review, however, the cost component was not considered and the focus was mainly on DALYs averted by these interventions. Based on this analysis, several factors were identified that affect the performance of these interventions in averting DALYs. The following six are some of the factors that need due consideration by programmers during the implementation of prevention or control activities at national or subnational levels: i) implementation coverage of the interventions; ii) degree of combination of multiple interventions across public health functions; iii) subsidization or providing free interventions; iv) implementation in constituencies of high disease burden or prevalence v) early identification and management of risk factors and vi) applicability of some interventions to specific age cohorts only;.

The coverage and combination of interventions across public health functions demonstrated a significant impact in averting DALYs. Interventions implemented at higher coverage were more effective in averting DALYs than those with low coverage. When ITN was implemented as an intervention for malaria control at 50% coverage, it averted 4,961,812 DALYs but averted 7,629,171 and 8,872,378 average yearly DALYs when coverage was increased to 80% and 95%, respectively [48]. Implementing multiple health promotive, preventive and curative interventions in combination was found to avert more DALYs than a single intervention. The provision of only pneumococcal vaccine for the prevention of lower respiratory tract infections in children averted 3,465,493 DALYs. In contrast, a combination of breastfeeding promotion, pneumococcal vaccine, Haemophilus influenzae type B vaccine, use of cleaner liquid fuels, community-based case management and zinc supplementation in combination averted 23,081,510 DALYs [29]. In road safety, the combined implementation of helmet use, safety belt use, and breath tests for alcohol were effective in averting more DALYs than the implementation of a single intervention independently. Similarly, interventions were more effective in improving newborn survival and averting newborn deaths when combined than when considered separately. The combination of multiple promotive and preventive interventions is particularly relevant in repivoting health systems towards UHC as the implication is instituting deliberate efforts to shift service delivery to strengthening primary care [124, 125].

Another important observation noted was the effectiveness of similar interventions applied with the same coverage may differ depending on disease burden or the population exposed to the risk of a particular condition. Case management with artemisinin-based combination therapy with 95% coverage averts 9,254,473 average yearly DALYs in West Africa, while the similar intervention with the same coverage averts only 5,886,159 average yearly DALYs in East Africa [48]. Effectiveness studies of some interventions primarily related to NCDs, have been performed in the context of high-income countries; thus, the question arises whether such studies apply to health care delivery settings in developing countries. For example, the higher the background incidence of a condition, the more effective a prevention or screening intervention is likely to be. Therefore, the relative incidence patterns for disease intervention in HICs and LMICs/LICs must be carefully considered. A good example is the relatively high incidence of cervical cancer in developing countries compared to developed countries. Interventions for cervical cancer prevention and screening are likely to be more cost-effective in developing countries than in developed countries, all else being equal. Therefore, the critical contributions varying contexts contribute to the effectiveness of different interventions, the invaluable role of determinants of health in contributing to health and well-being is underlined. In this regard, the social circumstances in a setting will influence service coverage, access and acceptability [126, 127].

The impact of price subsidies or provision of free interventions resulting in higher effectiveness was another important finding of this study. Price subsidies on fortified packaged infant cereals (F-PICs) were effective interventions for addressing dietary iron deficiency. It averted 268,301 DALYs at 50g/d when given free; averted 95,599 DALYs at 80% discount and 60,840 at 50% discount among individuals of poor socioeconomic status [28]. In the case of epilepsy and mental disorders, public financing for epilepsy treatment and the enhanced availability of medications demonstrated high effectiveness in averting the DALYs for these conditions [128]. In HIV prevention, sustained free HIV voluntary counselling and testing was more effective in averting DALYS than paid services. Relatedly, evidence has shown how using payment incentives supports positive patient and provider behaviour changes and how expanding insurance coverage opens up healthcare to many populations. Furthermore, social health determinants such as non-financial barriers related to indirect costs (e.g., food, fear of loss of income), also influence acceptability and access e.g., treatment with dignity and non-discrimination, and ought to be considered [129].

In preventing NCDs, combination drug treatment for individuals with a low absolute risk of a cardiovascular event was more effective in averting more DALYs than when treatment was given to individuals with high absolute risk. While the 35% absolute risk averts only 264 716 DALYs per year, combination drug treatment for individuals with a 5% absolute risk averts 404 684 DALYs per year, indicating the need for early risk detection and its management.

This review also identified that these interventions are not equally effective across all age cohorts as some of the interventions are limited to some population groups. In contrast, many others demonstrated effectiveness across the life course. Taking malaria-related interventions as an example, intermittent preventive treatment in infants (IPTi) and intermittent preventive treatment in pregnant women (IPTp) were some of the specific interventions that demonstrated effectiveness in infants and pregnant women, respectively. On the other hand, insecticide-treated bed nets (ITNs) and indoor residual spraying (IRS) were effective interventions for malaria control across all age cohorts. Regulations for tobacco use, road transport use and healthy diets such as salt, sugar and unhealthy fat consumption were interventions that demonstrated effectiveness across several age cohorts. Thus, the important role of multi-sectoral collaborations, both within and outside of the health sector, also influences effective coverage and needs to be closely coordinated and regulated [130]. The indirect effects of strengthened multi-sectoral collaborations have been shown to provide additional benefits of better health risk preparedness [131].

Most interventions across public health functions that averted more DALYs were easily implementable at community or lower levels of care. For example, condom promotion and Voluntary HIV counselling and Testing for HIV prevention, antiretroviral therapy for the management of HIV/AIDS were effective when implemented at the community and lower levels of care. Routine blood pressure and blood glucose screening and treatment for the prevention of cardiovascular diseases, and instituting school-based psychological interventions to prevent the onset of depression among students were effective interventions that could be easily implemented at the community and lower levels of care to avert thousands of DALYs. This means countries can improve access to effective interventions without causing unnecessary financial hardship to their population, especially those residing in remote areas. Most interventions assessed were effective; however, a few interventions showed low effectiveness. For example, Zehtab’s et al. [98], in analysing the cost-effectiveness of breast cancer screening using mammography in 35-69year-old women in an Iranian setting, found low effectiveness of annual breast cancer screening due to the short interval of the screening program. The focus on community-level interventions places communities at the centre, ensuring what the systems provide is aligned to population needs and that the care provided is person-centred. However, there need to be more efforts to ensure no groups are left behind, particularly the disadvantaged and that the equity lens used mirrors the social gradient and the complexity of social stratification of communities [132, 133].

Even though this study generated effective interventions for the different age cohorts and public health functions, ranking the interventions using the averted DALYs was difficult due to varied methodologies across studies. To allow comparison across the interventions and ranking of the most effective ones using DALYs, an extra step involving modelling all the identified interventions and standardizing the methodology, including assumptions, would be required. Therefore, further analysis using standardised methods would enhance the potential generalizability and applicability of the study findings to comparable settings within the African continent.

Our analysis of the burden of disease indicated that, despite the declining trends in CMNN conditions and increasing trend of non-communicable diseases in the African Region, neonatal conditions and communicable diseases remain the major contributors of DALYs. It also indicated that the burden distribution varies across the life course, with some conditions dominating specific age cohorts more than others. However, some conditions such as communicable diseases (lower respiratory tract infections, diarrheal diseases, malaria, HIV and tuberculosis) are not limited to specific age groups but remain challenges across several age cohorts. In the burden of disease study of 2019, neonatal disorders were the leading contributors (32%) of global DALY in the under ten age group. Our findings also confirmed that neonatal conditions remain the leading contributors of DALYs in neonates and early childhood. This was reflected in the high neonatal mortality rate of 27 deaths for 1000 live births in sub–Saharan Africa, making it the highest in the world [134]. Though the contribution of NCD DALYs in the African region was only 10%, three NCD conditions (cerebrovascular diseases, cancers and diabetes mellitus) were responsible for almost 35.5% of the DALYs among the cohort of elderly. Nonetheless, it should also be noted the contribution of communicable diseases to the development of NCDs and specifical cancers in the African region as up to one-fourth of cancers in developing countries are associated with chronic infections such as Hepatitis B Virus (HBV), Human Papillomavirus (HPV) and Helicobacter Pylori (H pylori) infections [123]. Hence, countries that strive to achieve universal health coverage need to focus their prioritization and resources on conditions that contribute to the largest contributors of DALYs.

The analysis of disease burden also showed that some conditions may not rank as leading causes of DALYs when considered individually but may cause significant disease burden and become important public health problems when grouped, further exposing risk for health security. Significant among these groups were maternal conditions, NTDs, vaccine-preventable and epidemic-prone diseases. According the disease burden data base, approximately 46.6% of the 196,471 global maternal deaths in 2019 were in the African Region. The four leading conditions (Maternal haemorrhage, abortion and miscarriage, hypertensive disorders, sepsis and infection were responsible for almost 54% of deaths that occurred in the region. With 542 deaths per 100 000 live births, sub-Saharan Africa has the highest maternal mortality ratio (MMR) in the world, but the main causes of death were not among the top contributors of DALYs in the region. Even though they didn’t present as leading causes individually, a group of 20 diseases designated by WHO as neglected tropical diseases (NTDs) [135] were responsible for an estimated 16.5 million DALYs in 2019, with over 39% of the DALYs contributed by the Africa region. Likewise, vaccine-preventable diseases such as whooping cough and measles were responsible for 9,676,707 DALYs in the cohort of early childhood. The impact of these conditions on the poor communities and their amenability to effective interventions that could lead to their elimination is a valid justification for countries to include them as priority diseases when aiming to achieve UHC and Health security. Similarly, conditions of public health concern such as sickle cell disorders and epilepsy fuel the disease burden disproportionately, with the poor and vulnerable population is most affected due to unavailability and inaccessibility of services. Some conditions such as Invasive Non-typhoidal Salmonella have shown an increasing trend of almost 40% over the years in late childhood; however, studies that measure the effectiveness of interventions using DALYs averted for this condition were not found. The inclusion of effective interventions for these conditions in a health benefits package, say instituting public financing for epilepsy treatment; mass drug administration for NTDs; vaccination of children, or scaling up mandatory newborn screening for sickle cell disorder; serves the purpose of UHC and Health security for most African nations. There are also diseases that tally low as the DALYs contributed is a small fraction of the total (Ebola, 195,394.11, 100% in Africa, Yellow fever, 249,956.6, 86% in Africa) that need due consideration as their impact is catastrophic when it happens. To ensure the soundness of their health security and prepare for any shock that could disrupt the health system, countries also need to consider epidemic-prone diseases such as Ebola, haemorrhagic fevers etc. in addition to the leading causes of DALYs in their prioritization.

### Limitations

The findings for this study were anchored on published articles of a high standard and with a defined methodology; therefore, there was a marked under-representation of publications for the elderly age cohort and for some public health functions like the rehabilitation and palliative services, especially in the African setting. These are important areas that future research directions and countries need to think about as we move toward re-pivoting health systems.

This review used DALYs as its measure of effectiveness, and the cost component of cost-effectiveness was not considered within the scope of this study. Cost-effectiveness evaluation of an intervention is critical as it gives a more accurate depiction of whether an intervention will be feasible within a context or not. The affordability of a selected effective intervention would require careful consideration within the African context and the availability of the recommended interventions. While some interventions may be effective, they may not be cost-effective and affordable for a recommendation. Hence a further review that examines the full economic evaluation of the evidence is highly recommended to ensure recommended interventions are both cost-effective and affordable.

Studies showing whether an intervention is effective seldom cover the entire potential beneficiary population, and service providers in the private sector are often not recorded. Most studies, especially in developing countries, were purely modelling studies. Whereas heuristic extrapolation is an important first analytical step, indicating the need for more direct and realistic studies of benefit would be derived from clinical evaluation studies of interventions, specifically tailored to developing countries’ needs and conditions, including controlled clinical trials where possible.

## Conclusion

The evidence from the data generated clearly indicates that disease burden distribution varies across the life course, with some conditions dominating specific age cohorts more than others. Future research would benefit in further deciphering this pattern across sub-regions and countries, for tailored and targeted analysis Despite an increasing trend in NCDs, infectious diseases and neonatal conditions remain the major overall DALY contributors in the African region. It also demonstrated that effective health promotion, disease prevention and curative interventions exist for the major conditions contributing to Africa’s disease burden across the life course. Whilst some interventions have already been implemented at scale, and others have great potential to achieve a significant reduction in disease burden when customised to the country’s unique setting.

Therefore, to benefit from these effective interventions and achieve the triple aims of UHC, health security and coverage of health determinants, national and regional authorities and planners need to clearly identify the major conditions across the life course and prioritize interventions according to population needs and characteristics. Furthermore, they need to mobilize resources and engage multiple health and health-related stakeholders for a combination of interventions across public health, achieve high coverages, strive to identify and manage risk factors early, prioritize high disease burden areas and high-risk populations for the greatest possible impact during the implementation phase. With this available evidence aimed at UHC, countries can design and customise effective health benefits packages and leverage each other’s resources based on their unique socio-economic, demographic, and geo-political standing to benefit their populations.

## Supporting information

S1 Checklist Preferred reporting items for systematic reviews and meta-analyses (PRISMA) checklist. (DOC)

S1 Data Burden of diseases for leading conditions by age cohort. (XLSX)

S1 Table Search strategy for electronic databases. (DOCX)

S1 File Included studies and quality appraisal sheets. (XLSX)

S2 File Effective interventions by age cohort. (XLSX)
