# Supplementary material for: High impact health service interventions for attainment of UHC in Africa: A systematic review
Source: PLOS Glob Public Health. 2022 Sep 23;2(9):e0000945. doi: 10.1371/journal.pgph.0000945 (PMC10021619; doi:10.1371/journal.pgph.0000945)
Supplement: S1 Table — (DOCX) [file pgph.0000945.s003.docx]

**S1 Table.** Search strategy for electronic databases

| **Condition** | **Health Function** | **Key words** |
| --- | --- | --- |
| CVD + DM | Promotion | Pregnan* OR expectant OR gravid OR newborn OR neonatal OR infan* OR toddler* OR children OR child OR adolescent* OR teenage* OR teen* OR adult* OR elderly OR geriatric OR older person* AND CVD aware* OR cardiovascular* aware* OR diabetes aware* OR DM aware* OR CVD community health* OR DM community health* OR diabetes community health* cardiovascular* community health* OR CVD IEC OR DM IEC OR advoc* CVD services AND DALY* OR Disability adjusted life year* OR DALY* averted OR Disabilit* averted |
|  | Prevention | Pregnan* OR expectant OR gravid OR newborn OR neonatal OR infan* OR toddler* OR children OR child OR adolescent* OR teenage* OR teen* OR adult* OR elderly OR geriatric OR older person* AND CVD prevention* OR cardiovascular* prevent* OR diabetes prevent* OR DM prevent* OR CVD screen* OR DM screen* OR diabetes screen* cardiovascular* screen* OR lifestyle modif* OR lifestyle prevent* DM OR lifestyle prevent* CVD OR newborn screen* CHD OR newborn pulse* congenital heart AND DALY* OR Disability adjusted life year* OR DALY* averted OR Disabilit* averted |
|  | Curative | Pregnan* OR expectant OR gravid OR newborn OR neonatal OR infan* OR toddler* OR children OR child OR adolescent* OR teenage* OR teen* OR adult* OR elderly OR geriatric OR older person* AND CVD second* prevention* OR cardiovascular* second* prevent* OR diabetes second* prevent* OR DM second* prevent* OR CVD treat* OR DM treat* OR diabetes treat* cardiovascular* treat* OR lifestyle therap* OR diabetic foot prevent* OR retinopathy prevent* OR stroke prevent* OR coronary heart prevent* OR CKD prevent* OR renal failure prevent* OR access to CVD medic* OR access to diabetes medic* OR health* knowledge on diabetes OR health* knowledge on Cardiovasc* OR taskshift* CVD OR taskshift* diabetes AND DALY* OR Disability adjusted life year* OR DALY* averted OR Disabilit* averted |
|  | Rehab + Palliat | Pregnan* OR expectant OR gravid OR newborn OR neonatal OR infan* OR toddler* OR children OR child OR adolescent* OR teenage* OR teen* OR adult* OR elderly OR geriatric OR older person* AND CVD tertiary* prevention* OR cardiovascular* tertiary prevent* OR diabetes tertiary prevent* OR DM tertiary prevent* OR CVD treat* OR DM rehab* OR diabetes rehab* cardiovascular* rehab* OR cardiac rehab* OR diabetic foot rehab* OR retinopathy rehab* OR stroke rehab* OR coronary heart palliat* OR CKD palliat* OR renal failure palliat* OR access to CVD palliat* OR access to diabetes community support* OR diabetes patient group* OR Cardiovasc* patient group* OR CVD psychosocial* OR diabetes psychosocial* AND QALY* OR Quality adjusted life year* OR QALY* gained OR Qualit* gained OR Qualit* improved |
| Cancer | Health promotion | Pregnan* OR expectant OR gravid OR fet* OR newborn OR neonatal OR infan* OR children OR child OR adolescen* OR teen* OR adult* OR elder* OR old* OR geriatric AND diet* OR exercise* OR weight* OR radiation OR alcohol OR tobacco OR nutritious food* OR reduc* OR exclusive breastfeeding OR exclusive breastfeeding education OR safe sex* OR cancer risk factors and prevention* OR health education OR sexual partners OR condom* AND DALY* OR DALY* averted OR disability adjusted life year* OR disabilit* averted |
|  | Prevention | Pregnan* OR expectant OR gravid OR fet* OR newborn OR neonatal OR infan* OR children OR child OR adolescen* OR teen* OR adult* OR elder* OR old* OR geriatric AND health education OR hep* B vaccination OR HPV vaccination OR human papillomavirus vaccination OR clinical breast examinations (CBE) OR breast ultrasound OR self-observance OR self-breast examination* OR screening OR VIA/VILLI OR cytology OR treatment of pre-cancerous lesions AND DALY* OR DALY* averted OR disability adjusted life year* OR disabilit* averted |
|  | Curative | Pregnan* OR expectant OR gravid OR fet* OR newborn OR neonatal OR infan* OR children OR child OR adolescen* OR teen* OR adult* OR elder* OR old* OR geriatric AND physical and clinical examination OR pain reliev* OR diet OR nutritional support OR admission OR management of opportunistic infections OR management of any underlying conditions OR screening OR PAP smear OR colscopic examination OR blood tests OR biopsy OR x-rays OR CT scan OR PET scan OR management of complications OR blood transfusions OR oxygen therapy OR chemotherapy OR surg* OR hysterectomy OR brachytherapy OR radiotherapy OR Intensive care unit services OR ICU OR counsel* OR lymphadenectomy AND DALY* OR DALY* averted OR disability adjusted life year* OR disabilit* averted |
|  | Rehab and Palliat | Pregnan* OR expectant OR gravid OR fet* OR newborn OR neonatal OR infan* OR children OR child OR adolescen* OR teen* OR adult* OR elder* OR old* OR geriatric AND support groups OR social marketing OR clinical psychology services OR specialist teams OR psychology* care OR physical rehabilitation OR prosthetics OR post-surgical treatment OR psycho social support OR palliative pain relief OR spiritual support AND QALY* OR Quality adjusted life year* OR QALY* gained OR Qualit* gained OR Qualit* improved |
| COPD+Asthma | Health promotion | Pregnan* OR expectant OR gravid OR newborn OR neonatal OR infan* OR toddler* OR children OR child OR adolescent* OR teenage* OR teen* OR adult* OR elderly OR geriatric OR older person* AND COPD community educ* OR COPD community awareness OR advoca* for COPD healthy environment OR advoca* for smoke reduc* OR COPD IEC OR COPD train community health workers OR law of tobacco OR law on smoke reduc* AND DALY* OR Disability adjusted life year* OR DALY* averted OR Disabilit* averted |
|  | Prevention | Pregnan* OR expectant OR gravid OR newborn OR neonatal OR infan* OR toddler* OR children OR child OR adolescent* OR teenage* OR teen* OR adult* OR elderly OR geriatric OR older person* AND COPD prevent* OR COPD early respirat* infection treatment OR COPD asthma treatment OR avoid* indoor smoke OR avoid* tobacco smoke OR prevent* COPD OR physical activity to prevent COPD OR healthy diet to prevent* COPD AND DALY* OR Disability adjusted life year* OR DALY* averted OR Disabilit* averted |
|  | Curative | Pregnan* OR expectant OR gravid OR newborn OR neonatal OR infan* OR toddler* OR children OR child OR adolescent* OR teenage* OR teen* OR adult* OR elderly OR geriatric OR older person* AND COPD treatment* OR COPD therap* COPD diagnosis OR COPD hosp* OR COPD treat* strategies OR vaccine for COPD OR physical activity to manage* COPD OR healthy diet to manage* COPD AND DALY* OR Disability adjusted life year* OR DALY* averted OR Disabilit* averted |
|  | Rehab and Palliat | Pregnan* OR expectant OR gravid OR newborn OR neonatal OR infan* OR toddler* OR children OR child OR adolescent* OR teenage* OR teen* OR adult* OR elderly OR geriatric OR older person* AND COPD rehab* OR COPD palliat* COPD pulmonary rehab* OR COPD physiotherapy OR COPD social support OR COPD home based OR COPD psychosocial OR COPD community* support AND QALY* OR Quality adjusted life year* OR QALY* gained OR Qualit* gained OR Qualit* improved |
| Road Injuries | Health promotion | Pregnan* OR gravid* OR fetus OR fetal OR newborn* OR neonat* OR infan* OR children OR child OR adolescen* OR teen* OR adult* OR elder* OR older AND road traffic injur* aware* OR road traffic accident* aware* OR road traffic crash* aware* OR road traffic injur* health promotion OR road traffic accident* health promotion OR road traffic crash community health worker* OR road traffic accident* community health worker* OR road traffic injury community health worker* OR road traffic accident* community health system* OR multi-sector* AND DALY* OR Disability adjusted life year* OR DALY* averted OR Disabilit* averted |
|  | Prevention | Pregnan* OR gravid* OR fetus OR fetal OR newborn* OR neonat* OR infan* OR children OR child OR adolescen* OR teen* OR adult* OR elder* OR older AND road traffic injur* prevention OR road traffic accident* prevention OR road traffic crash* prevention AND DALY* OR Disability adjusted life year* OR DALY* averted OR Disabilit* averted |
|  | Curative | Pregnan* OR gravid* OR fetus OR fetal OR newborn* OR neonat* OR infan* OR children OR child OR adolescen* OR teen* OR adult* OR elder* OR older AND road accident trauma injury diagnosis OR road accident trauma injury stabiliz* OR road accident emergency trauma care OR road accident emergency injur* care OR road accident emergency trauma management OR road accident emergency injur* management OR road accident trauma injur* care OR road accident trauma injur* treatment OR road accident trauma injur* management AND DALY* OR Disability adjusted life year* OR DALY* averted OR Disabilit* averted |
|  | Rehab and Palliative care | Pregnan* OR gravid* OR fetus OR fetal OR newborn* OR neonat* OR infan* OR children OR child OR adolescen* OR teen* OR adult* OR elder* OR older AND trauma palliat* OR injury palliat* AND QALY* OR Quality adjusted life year* OR QALY* gained OR Qualit* gained OR Qualit* improved |
| Mental Disorders | Health promotion | Pregnan* OR expectant OR gravid OR fet* OR newborn OR neonatal OR infan* OR children OR child OR adolescen* OR teen* OR adult* OR elder* OR old* OR geriatric AND physical exercise OR school program* OR socio-emotional learning program* OR obesity prevent* OR obesity control OR minim* sedentary screen time OR reduc* screen time OR early child* program* OR alcohol tax* AND DALY* OR DALY* averted OR disability adjusted life year* OR disabilit* averted |
|  | Prevention | Pregnan* OR expectant OR gravid OR fet* OR newborn OR neonatal OR infan* OR children OR child OR adolescen* OR teen* OR adult* OR elder* OR old* OR geriatric AND screening for depression OR screening for substance *use OR physical exercise OR folic acid OR phosphatidylcholine OR omega-3 fatty acids OR fish oil OR Vitamin* A and D OR school program* OR regulatory ban* of hazardous pesticide* AND DALY* OR DALY* averted OR disability adjusted life year* OR disabilit* averted |
|  | Curative | Pregnan* OR expectant OR gravid OR fet* OR newborn OR neonatal OR infan* OR children OR child OR adolescen* OR teen* OR adult* OR elder* OR old* OR geriatric AND training of health work* OR training HRH OR psychological treatment OR newer generation medic* OR legislation mental health OR financing mental health OR collaborative care OR physical exercise OR behavio*r* activation OR cognitive behavior*r* therapy OR CBT OR step Up OR strategies and tools embrace prevention with upstream programs OR home* care AND DALY* OR DALY* averted OR disability adjusted life year* OR disabilit* averted |
|  | Rehab and Palliative care | Pregnan* OR expectant OR gravid OR fet* OR newborn OR neonatal OR infan* OR children OR child OR adolescen* OR teen* OR adult* OR elder* OR old* OR geriatric AND cognitive behavior*r* therapy OR CBT OR inpatient rehabilitation for homeless persons OR physical exercise OR employment program* OR day occupational therapy program* OR community* inclusive development OR mental health palliat* AND QALY* OR Quality adjusted life year* OR QALY* gained OR Qualit* gained OR Qualit* improved |
| Epilepsy | *Literature was very scarce and so the string incorporated all health functions* | Pregnan* OR gravid* OR fetus OR fetal OR newborn* OR neonat* OR infan* OR children OR child OR adolescen* OR teen* OR adult* OR elder* OR older AND epilepsy aware* OR epilepsy health promotion OR epilepsy prevent* OR epilepsy diagnos* OR epilepsy treatment* epilepsy management* OR epilepsy palliati* AND DALY* OR Disability adjusted life year* OR DALY* averted OR Disabilit* averted |
| Sickle cell disease | Prevention | Pregnan* OR expectant OR gravid OR newborn OR neonatal OR infan* OR toddler* OR children OR child OR adolescent* OR teenage* OR teen* OR adult* OR elderly OR geriatric OR older person* AND sickle cell genetic counsel* OR sickle cell genetic testing OR sickle cell genetic counseling and testing OR SCD gene* testing OR SCD gene* counseling AND DALY* OR Disability adjusted life year* OR DALY* averted OR Disabilit* averted |
|  | Curative | Pregnan* OR expectant OR gravid OR newborn OR neonatal OR infan* OR toddler* OR children OR child OR adolescent* OR teenage* OR teen* OR adult* OR elderly OR geriatric OR older person* AND sickle* cell antenatal screening OR sickle* cell prenatal screening OR sickle cell newborn screening OR sickle cell test* OR sickle cell diagnosis OR penicillin prophylaxis OR sicle cell opportun* screening OR sickle cell antibiotic prophylaxis OR sickle cell vaccine OR hydroxyurea OR sickle cell blood transfusion OR sickle cell iron chelation OR sickle cell exchange transfusion OR sickle cell folate supp* OR sickle cell malaria* AND DALY* OR Disability adjusted life year* OR DALY* averted OR Disabilit* averted |
|  | Rehab | Pregnan* OR expectant OR gravid OR newborn OR neonatal OR infan* OR toddler* OR children OR child OR adolescent* OR teenage* OR teen* OR adult* OR elderly OR geriatric OR older person* AND sickle cell rehab* OR sickle cell complication* rehab* OR rehab* for sickle cell OR SCD rehab* AND DQALY* OR Quality adjusted life year* OR QALY* gained OR Qualit* gained OR Qualit* improved |
|  | Palliative | Pregnan* OR expectant OR gravid OR newborn OR neonatal OR infan* OR toddler* OR children OR child OR adolescent* OR teenage* OR teen* OR adult* OR elderly OR geriatric OR older person* AND SCD palliat* OR sickle cell palliat* OR palliat* care for SCD OR palliat* for sickle cell AND QALY* OR Quality adjusted life year* OR QALY* gained OR Qualit* gained OR Qualit* improved |

| **Condition** | **Health Function** | **Key words** |
| --- | --- | --- |
| **TB** | Health promotion | Tuberculosis OR TB AND Pregnan* OR expectant OR gravid OR newborn OR neonatal OR infan* OR toddler* OR children OR child OR adolescent* OR teenag* OR teen* OR adult* OR elderly OR geriatric OR “older person” AND “Health information” OR “health education” OR “Peer-based education” OR “School-based education” OR “Information Education Communication” OR “IEC materials distribution” OR “Mass media” OR “Social and behavioural change communication” OR BCC OR advocacy AND DALY* OR DALY* averted OR disability adjusted life year* OR disabilit* averted |
|  | Prevention | Tuberculosis OR TB AND Pregnan* OR expectant OR gravid OR newborn OR neonatal OR infan* OR toddler* OR children OR child OR adolescent* OR teenag* OR teen* OR adult* OR elderly OR geriatric OR “older person” AND “BCG” OR “Bacille Calmette-Guérin” OR “contact trac*” OR “isolation” OR “HIV test*” OR counsel* OR TPT OR “Tuberculosis preventive therapy” OR “Tuberculosis preventive treatment” OR “latent tuberculosis infection” OR LTBI OR “active case finding” OR ACF OR “passive case finding” OR PCF OR “Tuberculin Skin Test” (TST) OR PPD OR “purified protein derivative” OR IGRA OR “Interferon gamma release assay” |
|  | Curative | Tuberculosis OR TB AND Pregnan* OR expectant OR gravid OR newborn OR neonatal OR infan* OR toddler* OR children OR child OR adolescent* OR teenag* OR teen* OR adult* OR elderly OR geriatric OR “older person” AND “quality-assured microscopy” OR microscopy OR “rapid diagnostic test” OR (RDT) OR “chest x-ray” OR “CXR” OR “CAD” OR “computer assisted diagnosis” OR DOTS OR “DOTS-plus” OR “directly observed short course” OR VOT OR “video observed treatment” OR “self-administered treatment” OR SAT OR MDR OR XDR OR “extrapulmonary” OR “multidrug resistant” OR “extensive resistant” OR “family based” OR “community based” OR “first line” OR FL OR “second line” OR “SL” OR DST OR “drug sensitivity test” OR “sputum collection’ OR “sputum transport*” OR “gastric aspirate” OR stool OR decentralized OR centralized OR private OR nutrition OR ARV OR ART OR HAART OR cotrimoxazole OR prophylaxis OR complication OR surgic* OR hybrid OR model OR “adverse drug reactions” OR (ADRs) OR “finance* support” OR “nutrition support” OR long OR short OR “all oral” OR “LAMP” OR lipoarabinomannan OR loop-mediated isothermal amplification OR line-probe assay OR LPA OR RR-TB OR “INH-resistant” OR “Rifampicin resistant” OR Xpert OR “Xpert MTB/RIF” OR “people living with HIV” OR PLWH OR PLHIV OR “nucleic acid amplification test” OR NAAT AND DALY* OR DALY* averted OR disability adjusted life year* OR disabilit* averted |
|  | Rehab and Palliat | Tuberculosis OR TB AND Pregnan* OR expectant OR gravid OR newborn OR neonatal OR infan* OR toddler* OR children OR child OR adolescent* OR teenag* OR teen* OR adult* OR elderly OR geriatric OR “older person” AND “multi-disciplinary” OR “pulmonary exercise” OR rehabilitative AND Pain OR suffering OR relief OR opioid OR “non-opioid” OR spiritual OR palliative OR psychological OR stigma OR “end-of life” DALY* OR DALY* averted OR disability adjusted life year* OR disabilit* averted |
| **Malaria** | Health promotion | Malaria OR Mal* AND Pregnant OR expectant OR gravid OR newborn OR neonatal OR infant* OR toddler* OR children OR child OR adolescent* OR teenage* OR teen* OR adult* OR elderly OR geriatric OR “older person” AND “Malaria Health information” OR “Malaria health education” OR “Peer-based education” OR “School-based education” OR “Information Education Communication” OR “Mass media” OR BCC OR “malaria posters" OR behavioural intervention” AND DALY* OR DALY* averted OR disability adjusted life year* OR disabilit* averted |
|  | Prevention | Malaria OR Mal* AND Pregnan* OR expectant OR gravid OR newborn OR neonatal OR infan* OR toddler* OR children OR child OR adolescent* OR teenag* OR teen* OR adult* OR elderly OR geriatric OR “older person” AND “Intermittent preventive treatment of malaria in pregnancy” OR (IPTp) OR “Larvicid*” OR “intermittent preventive treatment of infants” OR (IPTi) OR “Seasonal malaria chemoprevention” OR (SMC) OR “long-lasting insecticidal nets” OR LLIN* OR “Larva source management” OR LSM OR “Chemoprophylaxis travellers” OR “Topical insect repellents” OR “Insecticide-treated clothing” OR “Spatial repellents” OR “mosquito coils” OR “Malaria Vaccine” OR (RTS,S) OR “Integrated vector management” OR “Intersectoral coordination” OR “insecticide resistance” OR “insecticide resistance” OR “Entomological surveillance” OR IRS OR “indoor residual spray” OR “Intermittent preventive treatment of child*” OR (IPTc) AND DALY* OR DALY* averted OR disability adjusted life year* OR disabilit* averted |
|  | Curative | Malaria OR Mal* AND Pregnan* OR expectant OR gravid OR newborn OR neonatal OR infan* OR toddler* OR children OR child OR adolescent* OR teenag* OR teen* OR adult* OR elderly OR geriatric OR “older person” AND “light microscopy” OR (LM) OR “rapid diagnostic test” OR (RDT) OR “glucose-6-phosphate dehydrogenase testing” OR “chloroquine” “uncomplicated malaria” OR “Artemisinin” OR “Parenteral” OR “severe malaria” OR “Primaquine” OR “Plasmodium Vivax” OR “Plasmodium falciparum” OR “Community-based ” OR “community health worker” OR “uncomplicated malaria” OR “Mass drug administration” OR (MDA)  OR (TACTs) OR “antimalarial medicines” OR “antimalarial drug resistance” OR “malaria surveillance” OR chemotherapy” OR “Active malaria case detection” OR ACD OR RCD OR  “screen-and-treat” OR “test-and-treat” OR “mass screen-and-treat” AND DALY* OR “DALY* averted “OR “disability adjusted life year*” OR disabilit* averted |
|  | Rehab and Palliative care |  |
| **Diarrheal disease** | Health promotion | Pregnan* OR expectant OR gravid OR newborn OR neonatal OR infan* OR toddler* OR children OR child OR adolescent* OR teenag* OR teen* OR adult* OR elderly OR geriatric OR “older person” AND “Health information” OR “health education” OR “Peer-based education” OR “School-based education” OR “Information Education Communication” OR “IEC materials” OR “Mass media” OR “behavioural change communication” OR BCC OR “posters" OR “behavioural intervention” AND DALY* OR “DALY* averted “OR “disability adjusted life year*” OR disabilit* averted |
|  | Prevention | Pregnan* OR expectant OR gravid OR newborn OR neonatal OR infan* OR toddler* OR children OR child OR adolescent* OR teenag* OR teen* OR adult* OR elderly OR geriatric OR “older person” AND Sanitation OR immunization OR vaccination OR “safe water” OR “clean water” OR hygiene OR “zinc supplementation” OR “zinc” OR “breast feeding” OR “exclusive breast feeding” OR “hand washing” AND DALY* OR “DALY* averted “OR “disability adjusted life year*” OR disabilit* averted |
|  | Curative | Pregnan* OR expectant OR gravid OR newborn OR neonatal OR infan* OR toddler* OR children OR child OR adolescent* OR teenag* OR teen* OR adult* OR elderly OR geriatric OR “older person” AND ORS OR “oral rehydration salt” OR “oral rehydration therapy” OR ORT OR “vitamin A” OR “supplementation” OR antibiotics OR “complementary feeding” OR “supplementary feeding” AND DALY* OR “DALY* averted “OR “disability adjusted life year*” OR disabilit* averted |
|  | Rehab and Palliative care |  |
| **HIV/AIDS** | Health promotion | Pregnan* OR expectant OR gravid OR newborn OR neonatal OR infan* OR toddler* OR children OR child OR adolescent* OR teenag* OR teen* OR adult* OR elderly OR geriatric OR “older person” AND “Health information” OR “health education” OR “Peer-based education” OR “School-based education” OR “Information Education Communication” OR “IEC materials” OR “Mass media” OR “behavioural change communication” OR BCC OR posters OR “behavioural intervention” AND DALY* OR “DALY* averted “OR “disability adjusted life year*” OR disabilit* averted |
|  | Prevention | Pregnan* OR expectant OR gravid OR newborn OR neonatal OR infan* OR toddler* OR children OR child OR adolescent* OR teenag* OR teen* OR adult* OR elderly OR geriatric OR “older person” AND Condom OR “female condom” OR “male condom” OR PrEP OR “Pre-exposure prophylaxis” OR PEP OR “Post-exposure prophylaxis” OR ARV OR HAART OR “highly active antiretroviral therapy” OR “blood safety” OR “universal precaution” OR “needle exchange” OR “TAP” OR “treatment as prevention” OR “prophylaxis” OR “opportunistic infection prevention” OR “infection prevention” OR PMTCT OR eMTCT OR “prevention of mother to child transmission” OR “elimination of mother to child transmission” OR “HST” OR “HIV testing services” OR VCT OR “Voluntary counselling and testing” OR HST OR “HIV self-testing” OR TPT OR “TB prevention therapy OR treatment” OR IPT OR “Isoniazid preventive therapy” OR “INH prophylaxis” OR “Voluntary medical male circumcision” OR VMMC AND DALY* OR “DALY* averted “OR “disability adjusted life year*” OR disabilit* averted |
|  | Curative | Pregnan* OR expectant OR gravid OR newborn OR neonatal OR infan* OR toddler* OR children OR child OR adolescent* OR teenag* OR teen* OR adult* OR elderly OR geriatric OR “older person” AND ARV OR HAART OR “highly active antiretroviral therapy” OR “opportunistic infection prevention” OR adherence OR CD4 OR “CD4 monitoring” OR “viral load” OR suppression AND DALY* OR “DALY* averted “OR “disability adjusted life year*” OR disabilit* averted |
|  | Palliative and rehabilitative | Pregnan* OR expectant OR gravid OR newborn OR neonatal OR infan* OR toddler* OR children OR child OR adolescent* OR teenag* OR teen* OR adult* OR elderly OR geriatric OR “older person” AND AND “multi-disciplinary” OR “pulmonary exercise” OR rehabilitative AND Pain OR suffering OR relief OR opioid OR “non-opioid” OR spiritual OR palliative OR psychological OR stigma OR “end-of life” DALY* OR DALY* averted OR disability adjusted life year* OR disabilit* averted |
| **LRTI** | Health promotion | Pregnan* OR expectant OR gravid OR newborn OR neonatal OR infan* OR toddler* OR children OR child OR adolescent* OR teenag* OR teen* OR adult* OR elderly OR geriatric OR “older person” AND “Health information” OR “Peer-based education” OR “School-based education” OR “Information Education Communication” OR “IEC materials distribution” OR “Mass media” OR “Social and behavioural change communication” OR BCC OR advocacy AND DALY* OR “DALY* averted “OR “disability adjusted life year*” OR disabilit* averted |
|  | Prevention | Pregnan* OR expectant OR gravid OR newborn OR neonatal OR infan* OR toddler* OR children OR child OR adolescent* OR teenag* OR teen* OR adult* OR elderly OR geriatric OR “older person” AND “Face mask” OR vaccine OR “social distancing” OR “physical distancing” OR “Pneumococcal vaccine” OR “influenza vaccine” OR “covid vaccine” OR “cloth mask” OR hygiene OR sanitation OR “hand wash*” AND AND DALY* OR “DALY* averted “OR “disability adjusted life year*” OR disabilit* averted |
|  | Curative | Pregnan* OR expectant OR gravid OR newborn OR neonatal OR infan* OR toddler* OR children OR child OR adolescent* OR teenag* OR teen* OR adult* OR elderly OR geriatric OR “older person” AND “Antibody test” OR “rapid diagnostic test” OR (RDT) OR “Antigen test” OR PCR OR Dexamethasone OR antibiotic* OR antiviral OR Oxygen AND DALY* OR “DALY* averted “OR “disability adjusted life year*” OR disabilit* averted |
|  | Rehab and Palliative care | Pregnan* OR expectant OR gravid OR newborn OR neonatal OR infan* OR toddler* OR children OR child OR adolescent* OR teenag* OR teen* OR adult* OR elderly OR geriatric OR “older person” “multi-disciplinary” OR “pulmonary exercise” OR rehabilitative AND Pain OR suffering OR relief OR opioid OR “non-opioid” OR spiritual OR palliative OR psychological OR stigma OR “end-of life” AND DALY* OR DALY* averted OR disability adjusted life year* OR disabilit* averted |
| NTD | Health promotion | Pregnan* OR expectant OR gravid OR newborn OR neonatal OR infan* OR toddler* OR children OR child OR adolescent* OR teenag* OR teen* OR adult* OR elderly OR geriatric OR “older person” AND “Health information” OR “Peer-based education” OR “School-based education” OR “Information Education Communication” OR “IEC materials distribution” OR “Mass media” OR “Social and behavioural change communication” OR BCC OR advocacy AND DALY* OR DALY* averted OR disability adjusted life year* OR disabilit* averted |
|  | Prevention | Pregnan* OR expectant OR gravid OR newborn OR neonatal OR infan* OR toddler* OR children OR child OR adolescent* OR teenag* OR teen* OR adult* OR elderly OR geriatric OR “older person” AND “Mass drug administration” OR “MDA" OR "integrated vector control activities" OR "vector control" OR "surgery” OR “antibiotics” OR "face washing" OR "environmental sanitation" OR "intensified case management" OR "case management" OR "safe water" OR "sanitation" OR "hygiene" OR "preventive chemotherapy" AND DALY* OR “DALY* averted “OR “disability adjusted life year*” OR disabilit* averted |
|  | Curative | Pregnan* OR expectant OR gravid OR newborn OR neonatal OR infan* OR toddler* OR children OR child OR adolescent* OR teenag* OR teen* OR adult* OR elderly OR geriatric OR “older person” AND “Mass drug administration” OR “MDA" OR "integrated vector control activities" OR "vector control" OR "surgery” OR “antibiotics” OR "face washing" OR "environmental sanitation" OR "intensified case management" OR "case management" OR "safe water" OR "sanitation" OR "hygiene" OR "preventive chemotherapy" AND DALY* OR “DALY* averted “OR “disability adjusted life year*” OR disabilit* averted |
|  | Rehab and Palliative care | Pregnan* OR expectant OR gravid OR newborn OR neonatal OR infan* OR toddler* OR children OR child OR adolescent* OR teenag* OR teen* OR adult* OR elderly OR geriatric OR “older person” AND “multi-disciplinary” OR “pulmonary exercise” OR rehabilitative AND Pain OR suffering OR relief OR opioid OR “non-opioid” OR spiritual OR palliative OR psychological OR stigma OR “end-of life” AND DALY* OR” DALY* averted” OR “disability adjusted life year*” OR disabilit* averted |
| Neonatal sepsis |  | Newborn OR neonate OR baby or infant) AND (Quality umbilical cord care OR Chlorhexidine antiseptic OR Sterility procedure in male circumcision OR Clean environment OR protection from health care associated infections OR Infection prevention and control (IPC) bundle OR IPC training OR Infection prevention and control training OR text message reminders OR alcohol hand rub, OR enhanced environmental cleaning OR weekly bathing of babies ≥1.5 kg with 2% chlorhexidine gluconate OR Immunization OR Focused health education of clinicians OR Hand washing OR Exclusive breastfeeding OR Safe disposal of medical waste OR Breastfeeding OR Optimal nutrition OR Education through instructional posters OR written guidelines OR Build systems capacity OR Limitation of invasive procedures OR Screening for maternal Group B streptococcus infection OR Family collaborative care)) AND “Years lived with disability” OR YLD OR “Years of life lost” OR YLL OR DALY* OR “DALY*averted” |
| Birth Trauma |  | Newborn birth trauma OR or birth injury OR neonatal birth trauma or injury OR baby birth trauma or infant birth trauma) AND (Antenatal screening OR Prenatal diagnosis based on three-dimensional ultrasound formulae OR ultrasound monitoring OR Improve prenatal care OR control risk factors OR intrapartum sonographic measurements of body weight OR clinical cephalon-pelvimetry OR Staff training and simulation exercises OR intrapartum sonographic measurements of body weight OR Institutional protocols and algorithms OR population-based multivariate statistical techniques OR coordinate prescriptive plan of action birth trauma protocol OR Zavanelli maneuver OR Symphysiotomy OR Woods maneuver OR Skillful use of a logical series of maneuvers OR appropriate episiotomy and OR vacuum technique OR graphical documentation with visualization of the exact placement of vacuum cup OR safe caesarean section OR follow-up management OR Prevention and/or correction of contractures OR occupational therapy serial splinting/casting OR encouraging normal development)) AND “Years lived with disability” OR YLD OR “Years of life lost” OR YLL OR DALY* OR “DALY*averted”) |
| Congenital anomalies |  | Birth Defects OR Congenital anomalies, OR chromosomal anomalies OR Genetic anomalies or Birth anomalies OR congenital defects OR Newborn abnormalities OR Birth abnormalities AND  Antenatal screening OR Preconception care OR Whole exome sequencing OR chromosomal microarray of prospective parents OR Mitochondrial transfer OR gene editing OR  Folic Acid supplementation OR Early newborn screening OR national level Community-Based Screening OR Early-life health investment OR improve social determinants of health OR  Birth spacing OR nutritional supplementation “Years lived with disability” OR YLD OR “Years of life lost” OR YLL OR DALY* OR “DALY*averted”) |
| Obstetric hemorrhage |  | “Obstetric haemorrhage” OR “haemorrhage in pregnancy” or “bleeding in pregnancy” OR “hemorrhage in pregnant women” OR “haemorrhage during pregnancy” OR “blood loss in pregnancy” AND “Obstetric Hemorrhage Protocols across Health Systems” OR “Safe use of misoprostol” OR “Prevention of Intimate partner violence” OR “Optimal maternal nutrition” OR “Regular ANC” OR “monitoring in pregnancy” OR “risk assessment” OR “Improving access to emergency obstetric care” OR “Coordination and delivery of quality emergency care” OR “Address Female genital mutilation” OR “Aspirin use with caution” OR “Vital signs monitoring Basic Emergency Obstetric Care” OR “Timely referral” OR “uterine arteries prophylactic occlusion balloon placement” OR prophylactic temporary abdominal aortic balloon placement OR Power Doppler examination OR intravenous fluid replacement OR blood transfusion OR “assess blood loss” OR “Assess for shock” OR “pregnancy-related OR hysterectomy” AND “Years lived with disability” OR YLD OR “Years of life lost” OR YLL OR DALY* OR “DALY*averted” |
|  |  | “Maternal sepsis” OR “maternal Infections” OR “Obstetric sepsis” OR “postpartum sepsis” OR “puerperal infection” OR “Obstetric infection” OR “postpartum sepsis” AND Management OR intervention* OR treatment OR prevention* or Strateg* OR “preventive strategies OR therapy” AND AND “Years lived with disability” OR YLD OR “Years of life lost” OR YLL OR DALY* OR “DALY*averted” |
| Unsafe abortion |  | “Unsafe abortion” OR “abortion complications” OR abortion OR “complicated abortion” OR “abortion-related complications” AND Management OR intervention* OR treatment OR prevention* or Strateg* OR “preventive strategies” OR therapy AND “Years lived with disability” OR YLD OR “Years of life lost” OR YLL OR DALY* OR “DALY*averted” |
| Hypertensive disorder of pregnancy |  | Eclampsia OR pre-eclampsia OR preeclampsia OR “hypertension in pregnancy” OR “high blood pressure in pregnancy” or “elevated blood pressure in pregnancy” OR “increased blood pressure in pregnancy” or “hypertensive pregnant women” OR “pregnant women with eclampsia” OR “eclampsia in pregnancy” OR eclampsia OR “expecting mothers” AND “Interventions or strategies” OR “best practices or treatment or therapy or program or management” AND “Years lived with disability” OR YLD OR “Years of life lost” OR YLL OR DALY* OR “DALY*averted”) |
